# Insights into the Mechanism of Action of Bactericidal Lipophosphonoxins

**DOI:** 10.1371/journal.pone.0145918

**Published:** 2015-12-30

**Authors:** Natalya Panova, Eva Zborníková, Ondřej Šimák, Radek Pohl, Milan Kolář, Kateřina Bogdanová, Renata Večeřová, Gabriela Seydlová, Radovan Fišer, Romana Hadravová, Hana Šanderová, Dragana Vítovská, Michaela Šiková, Tomáš Látal, Petra Lovecká, Ivan Barvík, Libor Krásný, Dominik Rejman

**Affiliations:** 1 Institute of Organic Chemistry and Biochemistry, Czech Academy of Sciences v.v.i., Flemingovo nám. 2, 166 10 Prague 6, Czech Republic; 2 Institute of Microbiology, Czech Academy of Sciences v.v.i., Vídeňská 1083, 142 20 Prague 4, Czech Republic; 3 Department of Microbiology, Faculty of Medicine and Dentistry, Palacký University Olomouc, Hněvotínská 3, 775 15 Olomouc, Czech Republic; 4 Department of Genetics and Microbiology, Faculty of Science, Charles University in Prague, Viničná 5, 128 43 Prague 2, Czech Republic; 5 TRIOS, Ltd., Zakouřilova 142, Prague 4, 149 00, Prague, Czech Republic; 6 University of Chemistry and Technology, Technická 5, 166 28 Prague, Czech Republic; 7 Division of Biomolecular Physics, Institute of Physics, Faculty of Mathematics and Physics, Charles University in Prague, Ke Karlovu 5, 121 16 Prague 2, Czech Republic; Purdue University, UNITED STATES

## Abstract

The advantages offered by established antibiotics in the treatment of infectious diseases are endangered due to the increase in the number of antibiotic-resistant bacterial strains. This leads to a need for new antibacterial compounds. Recently, we discovered a series of compounds termed lipophosphonoxins (LPPOs) that exhibit selective cytotoxicity towards Gram-positive bacteria that include pathogens and resistant strains. For further development of these compounds, it was necessary to identify the mechanism of their action and characterize their interaction with eukaryotic cells/organisms in more detail. Here, we show that at their bactericidal concentrations LPPOs localize to the plasmatic membrane in bacteria but not in eukaryotes. In an *in vitro* system we demonstrate that LPPOs create pores in the membrane. This provides an explanation of their action *in vivo* where they cause serious damage of the cellular membrane, efflux of the cytosol, and cell disintegration. Further, we show that (i) LPPOs are not genotoxic as determined by the Ames test, (ii) do not cross a monolayer of Caco-2 cells, suggesting they are unable of transepithelial transport, (iii) are well tolerated by living mice when administered orally but not peritoneally, and (iv) are stable at low pH, indicating they could survive the acidic environment in the stomach. Finally, using one of the most potent LPPOs, we attempted and failed to select resistant strains against this compound while we were able to readily select resistant strains against a known antibiotic, rifampicin. In summary, LPPOs represent a new class of compounds with a potential for development as antibacterial agents for topical applications and perhaps also for treatment of gastrointestinal infections.

## Introduction

The introduction of antibiotics in the middle of the twentieth century had a tremendous effect on public health and world economy. Now, more than half a century later, the need for novel antibiotics is becoming increasingly apparent. While a very limited number of new antibiotic classes have been introduced in the last 40 years, the dramatic increase of antibiotic resistance has significantly compromised the efficiency of currently available compounds. This reduction in the efficacy of antibiotic treatment poses an urgent medical and economical problem [1, 2].

Historically, the term antibiotic was used for antibacterial compounds produced by microorganisms. In the text we will use this term also for semi-synthetic and synthetic compounds exhibiting antibacterial properties. Antibiotics typically target specific biosynthetic processes in the bacterial cell, such as inhibiting cell wall synthesis (e. g. β-lactams), DNA synthesis (e.g. quinolones), RNA synthesis (e. g. rifampicin), and protein synthesis (e.g. aminoglycosides) [3–14].

Originally, the cytoplasmic membrane was not considered a genuine antibacterial drug target because of the potential for membrane-active compounds to severely damage the mammalian cell membrane [1, 15]. This view, however, is changing as more information is available on the function of antimicrobial defense peptides that target the membrane [16]. Moreover, membrane active compounds such as daptomycin [17] and telavancin [18] are already in clinical use. This new knowledge has brought the bacterial membrane to the fore as an attractive target for antibiotics that can be specific for bacteria [19]. Such compounds also offer good prospects that resistant strains against these compounds will be difficult to emerge [20].

Recently, we reported the synthesis of novel compounds termed lipophosphonoxins (LPPO) exhibiting significant antibacterial activity [21]. The general structure of lipophosphonoxins consists of four modules: (i) a nucleoside module, (ii) an iminosugar module, (iii) a hydrophobic module (lipophilic alkyl chain), and (iv) a phosphonate linker module that holds together modules (i)-(iii). We demonstrated that LPPOs were bactericidal against various Gram-positive species, including resistant strains like vancomycin-resistant *Enterococcus faecalis*. The minimum inhibitory concentration (MIC) values of the best inhibitors were in the 1–12 mg/L range while their cytotoxic concentrations against human cell lines were significantly above this range. However, their mode of action was unknown.

In this study we systematically explored possible targets of LPPOs and we identified the cell membrane as their site of action. We show that LPPOs localize to the bacterial cell membrane where they form pores and this results in cell death. We determine their physico-chemical properties. Finally, we assess their genotoxicity, effects on living organisms using the mice model, ability to cross the epithelial barrier, and chances that resistant strains will appear.

## Materials and Methods

### Determination of MIC values

Antimicrobial activity of the tested compounds against aerobic and facultative anaerobic bacteria was assessed using the standard microdilution method determining the minimum inhibitory concentration (MIC) [22, 23]. Disposable microtitration plates were used for the tests. The samples were diluted in brain heart infusion broth (Himedia) to yield a concentration range between 200 mg/L and 1.56 mg/L (in some cases, the lower concentration range was extended to 0.78 mg/L). The plates were inoculated with a standard amount of the tested microbe—the inoculum density in each well was equal to 10^6^ CFU/ml. The MIC was read after 24 hours of incubation at 35°C as the minimum inhibitory concentration of the tested substance that inhibited the growth of the bacterial strains.

The minimum bactericidal concentration (MBC) is characterized as the minimum concentration of the sample required to achieve irreversible inhibition, i.e. killing the bacterium after a defined period of incubation. The MBC was examined by the inoculation method. 1 μl was transferred from microplate wells with defined concentrations of the tested sample and inoculated onto the surface of blood agar (Trios, Czech Republic). The MBC was determined as the lowest concentration that inhibited the visible growth of the used bacterium.

Standard reference bacterial strains (*Enterococcus faecalis* CCM 4224, *Staphylococcus aureus* CCM 4223*)* from the Czech Collection of Microorganisms (CCM), Faculty of Science, Masaryk University Brno and *Streptococcus agalactiae*, *Bacillus subtilis*, methicillin-resistant *S*. *aureus* 4591, fluoroquinolone-(ciprofloxacin)-resistant *S*. *haemolyticus* 16568, vancomycin-resistant *E*. *faecium* VanA419/ANA and methicillin-resistant *S*. *epidermidis* 8700/B strains obtained from the culture collection of Department of Microbiology (Faculty of Medicine and Dentistry, Palacký University Olomouc) were tested. All tested microorganisms were stored in cryotubes (ITEST plus, Czech Republic) at -80°C.

Antimicrobial activity of the tested compounds against anaerobic bacteria was assessed using the tube dilution method. **DR5026** and **DR5047** were used at final concentrations of 100.0, 50.0, 25.0, 12.5, 6.25, 3.125, 1.56, and 0.78 mg/L in Brain heart infusion broth (TRIOS, Ltd.). Each tube with 1 ml of culture media with different concentration was inoculated with either *Clostridium perfringens* CCM 4435, *Bacteroides fragilis* CCM 4712 or *Peptostreptococcus anaerobius* strains in concentration according to McFarland scale (3x10^8^ CFU/mL) and incubated under anaerobic conditions at +35°C for 72 hours. Growth in tubes was tested after 24, 48 and 72 hours of cultivation. After 24, 48 and 72 hours of incubation, samples were taken from the tubes with no visual growth and from the tubes with growth by the highest concentration of agents, streaked out on Schaedler blood agar plates (TRIOS, Ltd.) and incubated under anaerobic conditions (80% N_2_, 10% CO_2_, 10% H_2_) at +35°C +/-2°C for 48 hours to confirm the results. The samples were streaked out at the same time on Columbia blood agar plates (TRIOS, Ltd.) and incubated under aerobic conditions at +35 +/-2°C for 24 hours, also for confirmation. Credible results were acquired after 48 hours incubation in tubes, and after 72 hours incubation the results were confirmed. This procedure was performed 2 times in 3 series with each antimicrobial agent.

### Inhibition of biosynthesis of selected macromolecules

#### Growth of cells

*B*. *subtilis* 168 [24] was streaked out on LB agar and incubated at 37°C overnight. The next day, two loopfuls of the cells were suspended in 200 μl of the MOPS defined medium [25]. A fraction of this suspension was inoculated into 15 ml of MOPS so that OD_600nm_ at time 0 min was 0.03. The bacterial culture was grown in a shaker at 37°C to an early exponential phase (OD_600nm_ ~ 0.26–0.3). At this point, the cells were labeled with radioactive molecules that were subsequently incorporated into the macromolecules of interest. The rate of incorporation was measured and served as the readout for the effects of the studied compounds. The experiments were conducted as described previously [26–30] with minor differences. The protocol is described in detail for RNA synthesis and for the subsequent experiments only the differences are described.

#### RNA synthesis

RNA was labeled with [^3^H]-uridine (1 μCi/1 ml) and cold uridine was added to a final concentration of 100 μM. After 5 min the cells were distributed in 4 tubes (2.5 ml per tube). Then, we added either **DR5026** (2 mg/L)**, DR5047** (4 mg/L), rifampicin (2 mg/L), or an appropriate amount of water as the negative control. At 5, 10, 20, and 30 min, 100 μl and 250 μl of cells were taken to measure the cell density and determine the radioactive content, respectively. The 250 μl were mixed with 1 ml of 10% TCA and allowed to sit on ice for at least 1 h. Then, each sample was vacuum filtered (Whatman GF/C glassfiber), washed twice with 1 ml of 10% TCA and 3 ml of ethanol. The filters were then dried, scintillation liquid was added and the radioactivity of the samples was measured. The signal was normalized to cell density (OD_600nm_).

#### DNA synthesis

DNA was labeled by [^3^H]-deoxythymidine (5 μCi/ml); cold deoxythymidine was added to a final concentration of 5 μM. The next steps were the same as for RNA synthesis. The only difference was that nalidixic acid (100 mg/L) was used as the positive control [26].

#### Protein synthesis

Proteins were labeled with [^35^S]-methionine (1 μCi/1ml); cold methionine was added to a final concentration of 100 μM. As the positive control, chloramphenicol was used at 10 μg/ml. Aliquots for subsequent analyses were taken at 10, 20, 30, 40, and 60 min, respectively [27].

#### Lipid synthesis

Cells (5 ml aliquots) were labeled with [^3^H]-sodium acetate (20 μCi/ml); cold sodium acetate was added to a final concentration of 50 μM. Cerulenin (100 mg/L) was used as the positive control. 500 μl aliquots of cells were mixed with 500 μl of ice-cold sodium acetate (0.5 M) in 2 ml test tubes and were allowed to sit on ice for at least 40 min. Then, 1 ml of carrier *B*. *subtilis* cells (OD_600nm_~1) were added to each test tube and the cells debris was spun down at 14,000 g for 20 min. The supernatants were discarded and the pellets were washed twice with 1 ml of ice-cold sodium acetate (0.5 M). Lipids of cell membranes were extracted with 0.5 ml of methanol-chloroform mixture (1:1). Insoluble material was removed by centrifugation at 14,000 g for 20 min. Organic solvent was evaporated overnight under a fume hood, dry extracts were mixed with scintillation liquid and radioactivity was measured. The signal was normalized to cell density (OD_600nm_) [28, 29].

#### Cell wall synthesis

Cells were labeled with 1.5 μl of [^3^H]-N-acetyl glucosamine (final concentration 1 μCi/ml); cold N-acetyl glucosamine was added to a final concentration of 100 μM. After 10 min of incubation with the labeled substrate the cell suspension was distributed in 5 falcon tubes (volume of each sample was 1.5 ml). Ampicillin (2 mg/L) was used as the positive control [30].

### Electron microscopy assays

*B*. *subtilis 168* was grown in the LB medium at 37°C to mid-log phase (OD_600nm_ ~0.4). Then, the cell suspension was divided into 4 flasks—10 ml each—and either the tested compounds, **DR5026** and **DR5047** (at final concentrations of 10 mg/L or 20 mg/L, respectively), or water were added. After 15 and 30 min of incubation with LPPO the cells were collected by spinning down (10,000 g, 5 min), the pellets were suspended in 200 μl of phosphate buffer and used for electron microscopy assays. Transmission electron microscopy (TEM) with negative staining technique was used to examine the cell morphology before and after treatment with LPPO. PTA (phosphotungstic acid H_3_PW_12_O_40_) and uranyl acetate were used to enhance the contrast between the background and bacteria. A drop of the bacterial suspension was placed on parafilm and covered with a carbon-parlodion-coated grid for several minutes. Then the samples were stained by 0.25% PTA (pH 7.3) or 1% uranyl acetate for 30 seconds, examined and photographed by using a JEOL JEM 1011 transmission electron microscope operated at 60kV.

### Localization of LPPOs within bacterial cells

*B*. *subtilis* was grown as described above. The disintegration and fractionation of the cells was performed as described [31]. Briefly, at OD_600nm_~ 0.3 the working suspension was divided into three flasks, 50 ml each, and **DR5026**, **DR5047** (final concentrations: 1 mg/L or 3 mg/L, respectively) or appropriate amount of water were added. After 10 min of incubation the cells were collected by spinning down (3,000 g, 10 min, SN1-supernatant, P1-pellet). The cells were washed twice by phosphate buffer (50 mM, pH 8.0) and resuspended in 10 ml of the same buffer containing lysozyme (300 mg/L) and 50 μl PMSF (100 mM in propanol). The samples of cell suspension were intensively mixed with DNAse, RNAse (10 mg/L). Then MgSO_4_ (10 mM) was added immediately and the samples were incubated at 37°C for 30 min. Lysis was stopped by adding of EDTA (final concentration 15 mM). After 1 min MgSO_4_ was added again to a final concentration of 20 mM. The cell debris was spun down at 3,000 g for 10 min (SN2, P2). The supernatant (SN2) which contained cell membranes was transferred into a new plastic tube and centrifuged at 25,000 g (30 min) to separate the plasma membrane fraction (P3, pellet) and cytosol (SN3, cytoplasm). Selected fractions were analyzed by HPLC and the peaks containing LPPOs were verified by MS.

### HPLC analysis

Supernatants were used directly for the assays. The samples from pellets—cell debris and plasma membranes—were dissolved in methanol-chloroform mixture (1:1), insoluble material was removed by centrifugation at 14,000 g for 20 min. All samples were analyzed on a HPLC system with diode array and single quadrupole mass detection (Waters) on Luna PFP column (100x4.6 mm, 3 μm; Phenomenex) under gradient reverse phase conditions as followed: 0–100% B in 5 minutes, 100% C in 15 minutes, held for 10 minutes (A = H_2_O with 25 mM NH_4_Ac, B = 50% MeCN with 25 mM NH_4_Ac, C = MeCN), flow rate = 1 ml/min. **DR5026** was used as a standard.

### Pull-out experiments

*B*. *subtilis* 168 from exponential phase of growth was pelleted and resuspended in buffer A (20 mM Tris-HCl pH 8, 150 mM KCl, 1 mM MgCl_2_, 1 mM DTT, 0.5 mM PMSF), sonicated 10 x 15 s with 1 minute pauses on ice, and centrifuged. 10 μg of LPPO **DR5690** (synthesis is described in S1 Supporting Information) was incubated with magnetic streptavidin-coated beads (Sigma-Aldrich) in 300 μl of buffer A for 1 h at 4°C. The beads with LPPO and “empty” beads were washed with buffer A and incubated with ~400 μg of the cell lysate for 1 h at 4°C. Subsequently, the beads were 4x washed with buffer A. Finally, the beads were resuspended in 10 μl of buffer A plus SDS sample buffer, heated for 5 min at 95°C with gentle shaking and the eluted proteins were detected by SDS-PAGE and Coomassie staining (SimplyBlue, Invitrogen).

### Vesicle preparation and leakage assay

#### Lipid extraction

Bacterial phospholipids were extracted with hexane/2-propanol (3:2, v/v) mixture from *B*. *subtilis* 168 cell biomass (2000 ml of culture in LB medium) harvested in exponential phase by rapid filtration (Synpor no. 5 filter, Pragochema, Czech Republic). Phospholipids from human erythrocytes (0.5 ml, volunteer blood donor) were extracted with chloroform/2-propanol mixture (7:11, v/v) after hemolysis using distilled water. After evaporation of the solvent *in vaccuo* at 40°C, the phospholipids were dissolved in chloroform, filtered through glass fiber filters (Whatman) and concentrated under a stream of nitrogen. The lipid samples were stored at -80°C and used for liposome preparation.

#### Vesicle preparation

Vesicles to be used in the carboxyfluorescein (CF) leakage assay were prepared by mixing the appropriate amount of lipids (1 mg/ml) with chloroform/methanol 2:1 (v/v). The solvent was subsequently evaporated under a nitrogen gas stream obtaining a thin film on the walls of a glass tube. The hydration procedure in a buffer containing 50 mM CF, 5 mM HEPES pH 7.4 lasted for 60 min at 40°C, interrupted by thorough vortexing to form multilamellar vesicles. Large unilamellar vesicles (LUV) were prepared by repeated extrusion of the multilamellar vesicles through 100 nm polycarbonate filters (Avestin) using the LiposoFast Basic apparatus (Avestin, Canada). Vesicles were separated from nonencapsulated dye by gel filtration on Sephadex G-50 using 100 mM NaCl, 0.5 mM Na_2_EDTA and 5 mM HEPES, pH 7.4 as elution buffer. Fractions with the highest content of the entrapped dye were pooled and dissolved in the same buffer to the final phospholipid concentration of 60 μM.

#### Leakage assay

The release of vesicle contents after adding LPPO solutions was evaluated by the increased fluorescence intensity of CF released into the milieu which we express as the percentage of leakage. As we found that LPPOs are potent permeabilizers of the bacterial membrane we used high concentrations of phospholipids (60 μM) and relatively low concentration of LPPO (0.8 mg/L) to obtain reasonably slow kinetics for precise analysis (half time of lysis ~ 60 s). Maximum release of CF fluorescence (*F*_max_) was induced by lysing the vesicles with 0.1% (v/v) Triton X-100. Fluorescence intensity was monitored in time (excitation at 480 nm, emission at 515 nm) at 25°C using FluoroMax-3 (Jobin Yvon, Horriba) spectrofluorometer. The following equation was used to calculate the percent of CF leakage: % CF leakage = [(*F*–*F*_0_)/(*F*_max_−*F*_0_)] × 100, where *F* is the actual fluorescence intensity and *F*_0_ is the fluorescence intensity before addition of the permeabilizing agent. Using Fityk software [32] the time courses of liposome lysis were fitted by a function: *α*(1-exp(-*t*/τ))^*n*^, where *t* is the time, *α* is the amplitude of the effect, *τ* is the time constant of the effect, and *n* is Hill coefficient (S3 Fig).

### Lipid bilayer experiments [33]

A Teflon chamber was divided into two compartments connected by a circular aperture of about 0.5 mm in diameter. Black lipid bilayer membranes were formed by painting a solution of 3% (w/v) asolectin (type IIS phosphatidylcholine; Sigma-Aldrich) in n-decane/butanol (9:1, v/v) across the hole. Both compartments contained 2 ml of 10 mM Tris, 1M KCl, pH 7.4. The temperature was kept at 25°C. Tested LPPO was added to the *cis* side of the membrane at a concentration of 5 mg/L. The membrane current was measured with Ag/AgCl electrodes (Theta) with salt bridges (applied voltage 45 mV, *cis* positive), amplified by LCA-200-100G or LCA-4k-1G amplifier (Femto) and digitized by KPCI-3108 card (Keithly). The signal was processed by QuB software [34]. The histograms of single pore conductance was calculated from at least 500 events using a 50 pS bin size and fitted with Gaussian functions using Fityk software [32].

### Membrane permeabilization assay

Bacterial cells (*B*. *subtilis* 168, *S*. *epidermidis* 8700/B, *E*. *faecalis* CCM 4224) grown aerobically in nutrient media (LB medium for *B*. *subtilis*, Mueller Hinton for *S*. *epidermidis*, and Brain-heart infusion for *E*. *faecalis*) at 37°C to mid log phase (OD_450nm_~ 0.5) were harvested (8000 g, 25°C, 10 min), washed, and resuspended (final OD_450nm_~ 0.2) in a buffer containing 10 mM HEPES (pH 7.2), 0.5% glucose and 10 μM propidium iodide (PI, Invitrogen). Tested compounds were added to 2 ml of bacterial suspension in 10 × 10-mm quartz cuvette and propidium iodide (PI) uptake into cells (indicating membrane permeabilization) was monitored as the increase in fluorescence intensity (excitation at 515 nm, emission at 620 nm with bandpass 5 and 10 nm, respectively) at 25°C using FluoroMax-3 (Jobin Yvon, Horriba) spectrofluorometer. We used optical filters for suppression of light scattered by the cells (Omega Optical filters 3RD500-530 and 3RD570LP in excitation and emission paths, respectively). Bacterial suspension was continuously stirred by magnetic stirrer during the measurements. As a positive control for cell permeabilization, 10 μM melittin (Sigma) was added to the cuvette whereas the buffer addition served as a negative control (baseline). Presented data are the recorded intensities without background subtraction.

Mouse macrophages J774A.1 (ATCC, number TIB-67) were cultured at 37°C in a humidified air/CO_2_ (19:1) atmosphere in RPMI medium supplemented with 10% (v/v) heat-inactivated fetal bovine serum, penicillin (100 IU/ml), streptomycin (100 mg/L), and amphotericin B (250 μg/L). For fluorescence measurements, cells were mechanically harvested, seeded on glass coverslips in 6-well plates, and grown to 30% confluence (about 2x10^5^ cells). Cells grown on coverslips were washed in modified HBSS solution (140 mM NaCl, 5 mM KCl, 2 mM CaCl_2_, 3 mM MgCl_2_, 10 mM HEPES-Na, 50 mM glucose, pH 7.4) and promptly mounted into custom-made polypropylene holder which was placed horizontally into a quartz cuvette filled with HBSS containing 10 μM PI. The uptake of PI was observed as fluorescence increase using identical setting as with bacterial cells (see above). The observed area of the coverslip was about 10 mm^2^, corresponding to approximately 10^4^ cells.

### Interaction of fluorescently labeled LPPO DR5823 with membrane

The excitation spectrum (250–400 nm) and emission spectrum (340–600 nm) of **DR5823** (Synthesis described in S1 Supporting Information) were recorded after 10 minutes of incubation in buffer (100 mM NaCl, 0.5 mM Na_2_EDTA, 5 mM HEPES, pH 7.4) and in LUVs (100 nm, 100 μM DOPC, Avanti Polar Lipids) prepared as described above (for details see section Vesicle preparation and leakage assay). The wavelengths of 414 nm and 320 nm (5 nm bandpass) were used for excitation and emission, respectively. The background intensity of buffer and liposomes was subtracted from the recorded spectra.

The kinetics of **DR5823** membrane incorporation (observed as increase in fluorescence intensity) was recorded with 1 s integration time with the monochromator setting specified above. For the quenching experiment we added 120 μl of 5 M KI into 2 ml of liposome suspension yielding 283 mM final KI concentration. The intensity of fluorescence was corrected for the dilution of the sample.

### Molecular dynamics simulations

For details on MD simulations see S2 Supporting Information.

### Physicochemical properties and stability of LPPOs

Critical micellar concentration (CMC) values were measured (using conductometry method, COND 7, XS Laboratories instrument) and calculated according to literature procedure [35]. LogD values were calculated using software package ACD Labs [36].

Stability experiments were carried out at two concentrations and three pH values. Concentrations were 500 μmol/l (50 μl of 10 mM stock solution were dissolved in buffers of specific pH to a final volume of 1 ml) and 50 μmol/l (10 μl of stock solution were dissolved in buffers of specific pH to a final volume of 2 ml); pH: 0.18 (0.5 M HCl), 5 (0.1M NH_4_Ac adjusted with acetic acid), and 8.8 (0.1M NH_4_Ac adjusted with NaOH). The experiments were conducted at room temperature and 37°C, respectively. Samples were injected onto HPLC machine at different time points to assess the levels of decomposition. Time points: 0.5; 1; 3.5; 24 hrs. HPLC analytical parameters: Method: 0–100% B in 5 min, 100% C in 15 min, hold to 20 min. A = 25 mM NH_4_Ac, B = 25 mM NH_4_Ac in 50% MeCN, C = MeCN. Column: PFP, 100 x 4.6 mm, 3 um. Injection: 10 μl for 500 μM. Injection: 50 μl for 50 μM. Detection: PDA 210–400 nm; MS TIC 200–800 m/z.

### Ames test for genotoxicity

The Salmonella/microsome reversion assay was conducted using the plate incorporation procedure described by Marona and Ames [37]. The Ames test was performed utilizing two different bacterial strains of *Salmonella typhimurium*: strain TA98 for frame-shift mutations and strain TA100 for base-pair substitution. All strains were maintained and stored according to the standard procedures [38]. All samples were tested without metabolic activation (S9) to detect direct mutagenic compounds. The test was performed with plate incorporation method in duplicate at the increasing concentrations. The plates were incubated at 37°C for 72 h in the dark. The number of his+ revertants was estimated with the counter. Results were expressed as mutation ratios (Φ) calculated as the number of colonies on test plates/number of colonies on solvent control plates. A compound is considered mutagenic when Φ is more than 2 and a dose–response effect is evident [39].

### Transepithelial transport of LPPOs in Caco-2 monolayers

Transport of the tested compounds across the Caco-2 monolayers was studied using the BD BioCoat HTS Caco-2 assay system (BD Biosciences, Bedford, MA) according to the manufacturer’s instructions. The cells were seeded into the wells of the transwell insert at a concentration of 2 x 10^5^ cells per insert in the seeding basal medium. The cells were left to attach for 48 h, and after that the medium was changed to the enterocyte differentiation medium in which the cells were cultured for the next 48 h. Immediately before the transport experiments, the monolayers were washed twice by transport medium (HBSS containing 10 mM HEPES and 25 mM glucose, pH 7.4) and incubated for 30 min in a CO_2_ incubator at 37°C. The integrity of the cell monolayers was assessed by monitoring the transepithelial electrical resistance (TEER) using a Millicell ERS apparatus (Millipore, Bedford, MA) and paracellular flux marker, [^14^C]mannitol (0.15 μCi/insert). Intact cell monolayers displaying a TEER of > 250 Ω·cm^2^ were used for the experiments. The transport was initiated by the addition of 1 ml of the transport medium to the acceptor (basolateral) side and 300 μl of the transport medium containing 100 μM prodrugs to the donor (apical) side. After a 3 h incubation, fluids were collected from the donor and acceptor compartment and lyophilized. Cell monolayers were washed twice with ice-cold PBS. After that, for each compound, cells from two wells were solubilized using ice-cold cell lysis buffer (25 mM Tris, 150 mM sodium chloride, 1% Triton-X 100, pH 7.4) and scraped into 1.5 ml microcentrifuge tubes. Cell lysates were homogenized using a needle and syringe then centrifuged at 18,000 x g for 10 min. Finally, they were deproteinated by methanol precipitation, evaporated to dryness and together with donor and acceptor lyophilizates subjected to subsequent analysis.

The apparent permeability coefficient (Papp) was calculated from the following equation: Papp = (dQ/dt)/C0 x A, where dQ/dt is the rate of absorption of the drug across the cells, C0 is the donor compartment concentration at time zero, and A is the area of the monolayer.

Percent recovery was also calculated to reveal problems with poor solubility, binding of the compound to the plate or accumulation of the compound in the cell monolayer:

% recovery = 100 x (Cd + Ca)/C0, where Cd and Ca represent total concentrations of compound in donor and acceptor compartment, respectively, at the end of experiment. C0 is the donor compartment concentration at time zero.

#### Measurement

Donor (apical) and acceptor (basolateral) sites were lyophilized, dissolved in 150 μl water and filtered through 0.45 μm nylon filter that had been soaked with methanol. Cell lysate was extracted with 150 μl MeOH/CHCl_3_ (1:1) and filtered.

#### Analytical parameters

Method: 50–100% B in 5 min, 100% C in 12 min, hold to 20 min. A = 25 mM NH_4_Ac, B = 25 mM NH_4_Ac in 50% MeCN, C = MeCN. Column: PFP, 100 x 4.6 mm, 3 μm. Injection: 20 μl.

### MTD test in mouse model

The MTD (maximum tolerated dose) in mouse model was carried out by MediTox s.r.o. on a commercial basis. The complete protocol for the MTD testing can be found in S3 Supporting Information.

#### Preparation of the test item application formulations

For the administration the appropriate amount of the Test item (TI) was dissolved in water (aqua for injection, B. Braun, Germany) and administered to mice either orally (in the volume of 0.5 mL/25 g of body weight) or intraperitoneally (in the volume of 1.0 mL/25 g of body weight).

#### Test system

S3 and S4 Tables summarize the test system parameters, the number of animals used, and the doses tested. Briefly, for each dose, 3 males are 3 females were used.

#### DAILY clinical observations

All animals from the study were observed daily for clinical signs, morbidity or mortality during acclimation, 0.5, 4, 6, 24 and 48 hours after administration. Onset, duration and severity of any signs were recorded, and used as criteria for early euthanasia decisions. The clinical observations included: changes in skin, fur, eyes, occurrence of secretion and excretion; autonomic activity (e.g. lacrimation, piloerection); changes in gait and posture; changes in unprovoked behavior, presence of stereotypes or bizarre behavior; response to handling. Ether was used for anesthesia in compliance with the Standard Operating Procedures (SOPs) of MediTox, the European convention for the protection of vertebrate animals used for experimental and other scientific purposes (ETS 123), the Czech Collection of laws No. 246/1992, inclusive of the amendments, on the Protection of animals against cruelty, and Public Notice of the Ministry of Agriculture of the Czech Republic, Collection of laws No. 419/2012 as amended, on keeping and exploitation of experimental animals. MediTox s.r.o. is a holder of the accreditation Certificate for user’s issued by Central Committee for Animal Protection of the Czech Republic.

#### Terminal observations

All the animals that died during study or survived to their scheduled euthanasia (cervical dislocation under ether anesthesia) 8th day after the administration received a complete post-mortem examination and microscopic examination of organs showing evidence of gross pathology. The cause of death of animals that died during the study before the scheduled euthanasia was mainly venostasis of kidneys and liver.

### Selection for resistant strains

**A) *B*. *subtilis*.** *B*. *subtilis* 168 was incubated in the defined MOPS medium [40] with subcytotoxic concentrations of either rifampicin (starting at 0.01 mg/L; MIC 0.06 mg/L) or **DR5026** (0.5 mg/L; MIC ~ 3 mg/L) and grown at 37°C for 24 h. Then, the growth of the culture was evaluated by OD_600nm_ and an aliquot of the culture was transferred to a new tube with fresh medium and a two-fold increased concentration of the active compound. This template was followed until no growth was detected for **DR5026** or until *B*. *subtilis* grew at 5 mg/L of rifampicin.

**B) *Enterococcus faecalis* and *Streptococcus agalactiae*.** Induction of resistance was performed in microtitration plates by repeated exposure of bacterial strains to subinhibitory concentrations of tested substances (LPPOs). Samples were diluted in brain-heart infusion (BHI; HiMedia) exponentially and inoculated with the *E*. *faecalis* CCM 4224 (Czech collection of microorganisms, Masaryk University Brno) and *S*. *agalactiae* (the culture collection of Department of Microbiology, Faculty of Medicine and Dentistry, Palacký University Olomouc) strains. The final concentration of bacterial inoculum in the well was 10^6^ CFU/ml. Incubation was carried out at 35°C for 24 h. After incubation, 1 μl of BHI from wells with subinhibitory concentrations of tested samples were subcultured on blood agar (Trios, Ltd) for 24 h at 35°C. The obtained grown bacteria were used for the next step of resistance induction. The described procedure was considered to be one induction step. In total, 14 steps were made. Following each induction step, MICs of the tested substances were determined for the *E*. *faecalis* CCM 4224 and *S*. *agalactiae* strains.

## Results

### Compounds used in the study and selection of model organisms

**Fig 1:**
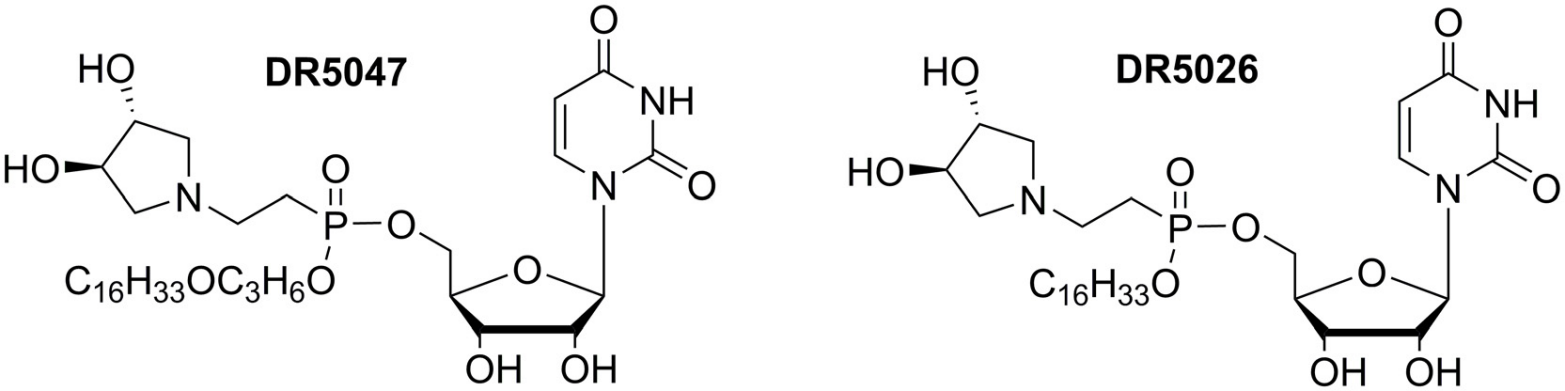
Structures of selected lipophosphonoxins DR5047, DR5026.

For the experiments presented in this study we used the two most potent compounds identified previously, **DR5047**, **DR5026** (Fig 1) [21]– **12b** and **12i,** respectively, in the original article. Table 1 summarizes their antibacterial activities.

**Table 1:** Antibacterial activity of lipophosphonoxins DR5047, DR5026.

	MIC mg/L	
	DR5047	DR5026
*Enterococcus faecalis* CCM 4224	6.25	3.125
*Staphylococcus aureus* CCM 4223	>200	25
*Bacillus subtilis*	6.25	3.125
*Streptococcus agalactiae*	6.25	3.125
*S*. *aureus* MRSA 4591	>200	25–50
*Staphylococcus haemolyticus* 16568	>200	50
*Enterococcus faecium* VanA 419/ana	6.25	3.125
*Staphylococcus epidermidis* 8700/B	3.125	3.125
*Clostridium perfringens*	12.5	12.5
*Bacteroides fragilis*	6.25	12.5
*Peptostreptococcus anaerobius*	1.56	1.56

For the experiments addressing the mode of action of LPPOs we selected *Bacillus subtilis*, a well-studied model Gram-positive soil bacterium against which both the selected LPPOs are active. As *B*. *subtilis* is a non-pathogenic species, we performed the key experiments also with clinically relevant species, such as *E*. *faecalis*, *S*. *epidermidis*, and/or *S*. *agalactiae*.

### Effect of LPPOs on biosynthesis of principal macromolecules

First, we tested the effect of **DR5047** and **DR5026** on the biosynthesis of DNA, RNA, protein, cell wall, and lipids. We first tested a series of concentrations of LPPOs to identify the highest concentration at which LPPOs did not cause a rapid decline in the cell density to be able to detect a potential effect on the synthesis of the aforementioned macromolecules. The identified concentrations were slightly below the respective MICs for these compounds; concentrations that were higher lead to rapid declines in cell density and would have likely had non-specific adverse effects on the synthesis of macromolecules.

**Fig 2:**
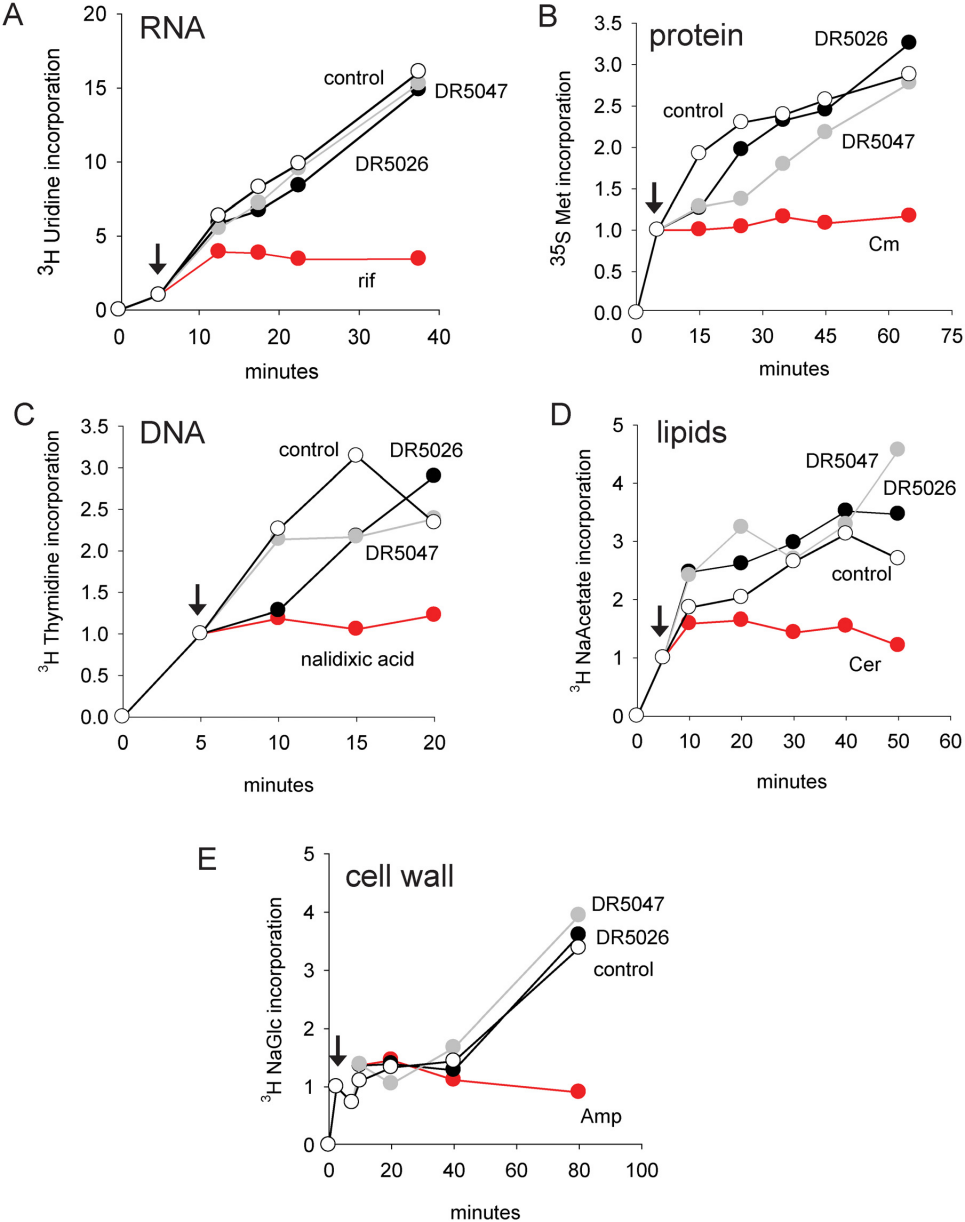
The effect of LPPOs on the biosynthesis of selected macromolecules. In all panels, **DR5026** is shown with black circles, **DR5047** grey circles, and control (no compound added) empty circles. The red symbols depict the effect of a known inhibitor. The amount of the radiolabeled material incorporated at the time of inhibitor addition (shown with arrows) was set as 1. **A.** The effect on RNA synthesis. Rif, rifampicin. **B.** The effect on protein synthesis. Cm, chloramphenicol. **C**. The effect on DNA synthesis. **D**. The effect on lipid synthesis. Cer, cerulenin. **E**. The effect on cell wall synthesis. Amp, ampicillin. The experiments were conducted in three biological replicates. Representative experiments are shown. The error was below 10%.

We grew *B*. *subtilis* to mid-exponential phase and then added a radiolabeled precursor for the biosynthesis of the macromolecule of interest (e. g. ^3^H uridine for RNA; for details see Mat&Met). We then distributed the culture into several aliquots, one of which was allowed to grow without the addition of any compound, and to the others we added one or the other LPPO or an antibiotic known to inhibit the synthesis of the particular macromolecule (e. g. rifampicin that binds to RNAP and stops transcription of DNA into RNA;) [41]. We subsequently withdrew aliquots at time points and determined the amount of the radioactive label being incorporated into the macromolecule. As can be seen in Fig 2, the two LPPOs did not significantly affect the biosynthesis of any of the macromolecules while known inhibitors were able to do so.

### Effect of LPPOs on the cell integrity

**Fig 3:**
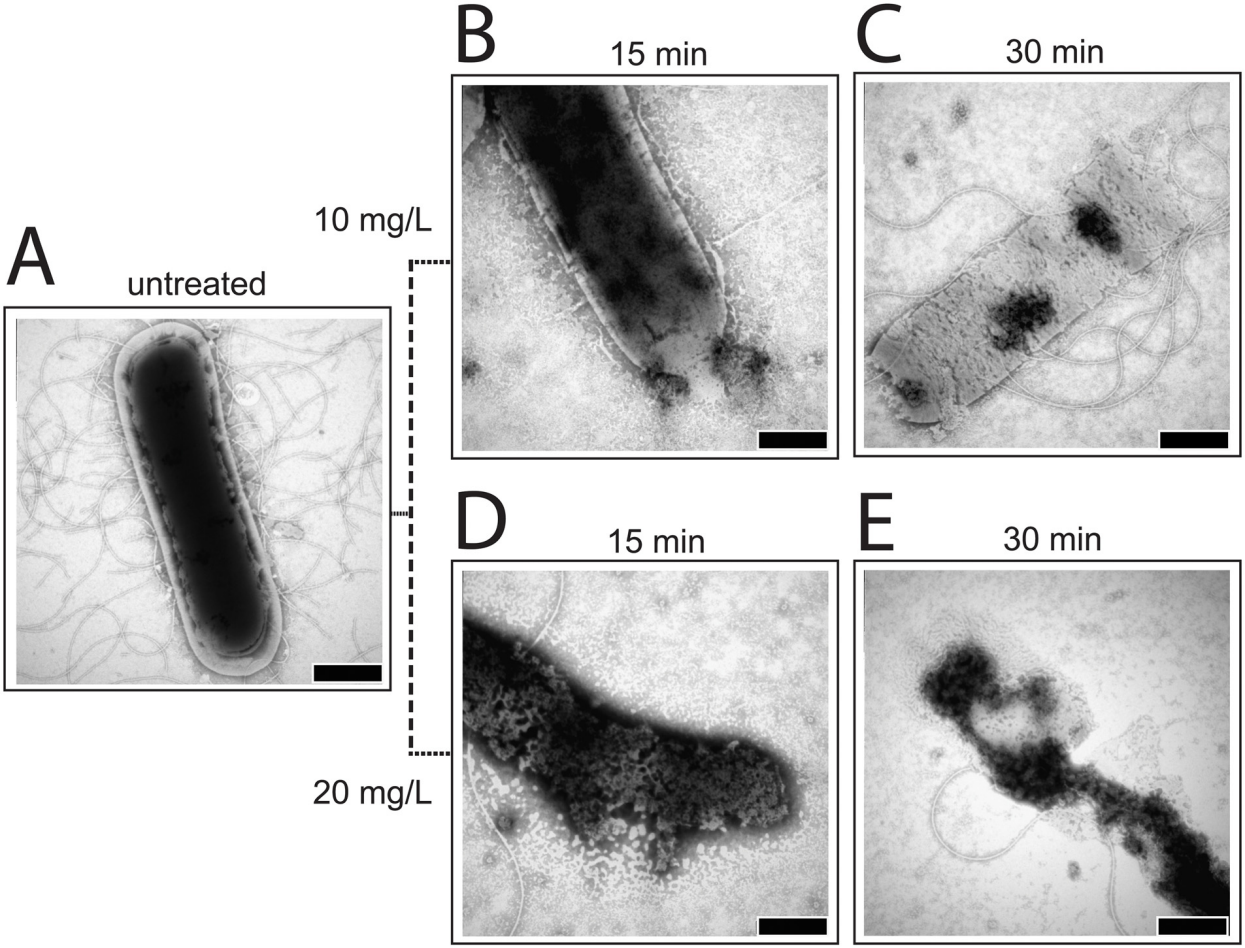
TEM pictures of *B*. *subtilis* cells. 0.25% phosphotungstic acid at pH 7.3 was used for staining. **A.** Untreated. **B**. Treated with 10 mg/L of **DR5026** for 15 min. **C**. Treated with 10 mg/L of **DR5026** for 30 min. **D**. Treated with 20 mg/L of **DR5026** for 15 min. **E.** Treated with 20 mg/L of **DR5026** for 30 min. The scale bars in the right-hand corners of the pictures represent 500 nm.

As LPPOs did not target the biosynthesis of any of the tested macromolecules, we decided to use electron microscopy to gain insights into the potential mode of function of these compounds. We treated the cells with LPPOs or water and withdrew aliquots after 15 and 30 min, respectively. The cells were then used for electron microscopy assays. Transmission electron microscopy (TEM) with negative staining technique was used to examine the *B*. *subtilis* cell morphology before and after the treatment with LPPOs. Fig 3 shows that the addition of either LPPO resulted in a major damage to the integrity of the cell. The cells appeared to be leaking the intracellular material to the outside, and after the longer incubation with LPPOs the cells even completely disintegrated. The leaking and disintegration were suggestive of an effect on the cytoplasmic membrane.

### Localization of LPPOs in bacterial cell and search for their potential metabolites

**Fig 4:**
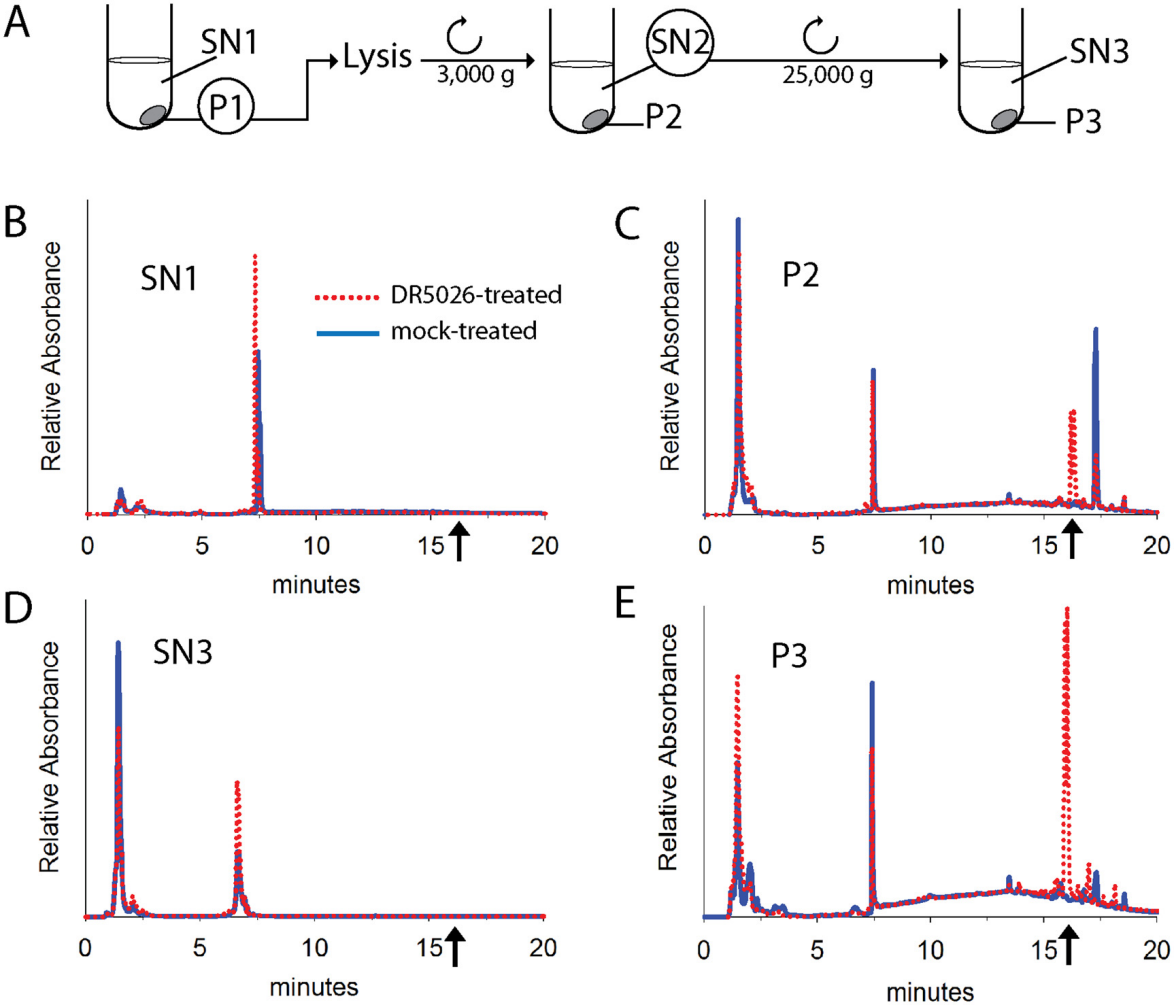
Localization of DR5026 in *B*. *subtilis* cells. **A**. A scheme of the experiment. SN, supernatant; P, pellet. **B.** HPLC data of supernatant after cell sedimentation, SN1. **C.** HPLC analysis of cell debris and remaining non-lysed cells, P2. **D.** HPLC analysis of cell cytoplasm, SN3. **E.** HPLC analysis of the plasma membrane fraction, P3. The dotted red line: **DR5026** treated cells; the blue line: mock-treated cells. The arrows indicate where **DR5026** eluted from the column. The identity of **DR5026** was confirmed by MS detection.

Subsequently, we investigated where LPPOs localize in the cell—i. e. whether they solely associate with the membrane or are also released to the cytosol. We used both bacterial (*B*. *subtilis*) and eukaryotic humans cells (cell line K526). We treated the cells with **DR5026** or **DR5047** for 10 minutes. A scheme of the experiment is shown in Fig 4A. Then we collected the cells by centrifugation (pellet, P1; supernatant, SN1). We lysed the cells and after subsequent centrifugation separated cell debris in pellet (P2, non-disrupted cells, cell wall fragments) from the cytosol and plasma membranes in supernatant (SN2). SN2 was then centrifuged at a high g to separate the cytosol (in supernatant, SN3) from plasma membranes (P3, in pellet). The fractions were analyzed by HPLC and the identity of LPPO was determined by mass spectrometry (MS). Fig 4 shows data for **DR5026**. In samples with bacterial cells, no LPPOs were found in SN1 (i. e. in the medium after treating the cells with **DR5026**; Fig 4B). It seemed that after the 10 min incubation all added LPPOs were captured by the cells. The cell debris contained LPPOs as it likely contained also some non-disrupted cells (Fig 4C). The cytoplasmic fraction (**SN3**) contained no LPPOs (Fig 4D) but LPPOs were found in the plasma membrane fraction (P3, Fig 4E). No detectable amounts of potential metabolites were found in the case of **DR5026** and only a small amount of **DR5047** was detected to be monoacetylated (< 5%; S2 Fig). In samples with eukaryotic cells (K562 cell lines), LPPOs were found in the first supernatant (i. e. in the medium) and not in the membranes or in the cytoplasm (data not shown), in correlation with previous results that demonstrated that LPPOs were not toxic to human cell lines at their bactericidal concentrations.

### Search for a potential protein binding partner(s) of LPPO

To test whether LPPOs, while in the membrane, perhaps also interact with a protein we performed pull-out experiments. We prepared a LPPO (based on the structure of **DR5026**) attached to magnetic beads. To do so, a triple bond containing LPPO **DR5557** (Scheme A in S1 Supporting Information) was synthesized and connected to biotin through azido group via click chemistry to yield biotinylated LPPO **DR5690** (Scheme B in S1 Supporting Information). Finally, the obtained biotinylated LPPO **DR5690**, was attached to streptavidin coated magnetic beads (Scheme C in S1 Supporting Information). The biotinylated LPPO (**DR5690**) still retained some antibacterial activity (S1 Table). Using cell lysate generated by sonication we were unable to detect any protein specifically interacting with **DR5690**, indicating that LPPOs may not require protein binding partners for their action (S1 Fig).

### LPPOs induce leakage from phospholipid vesicles

**Fig 5:**
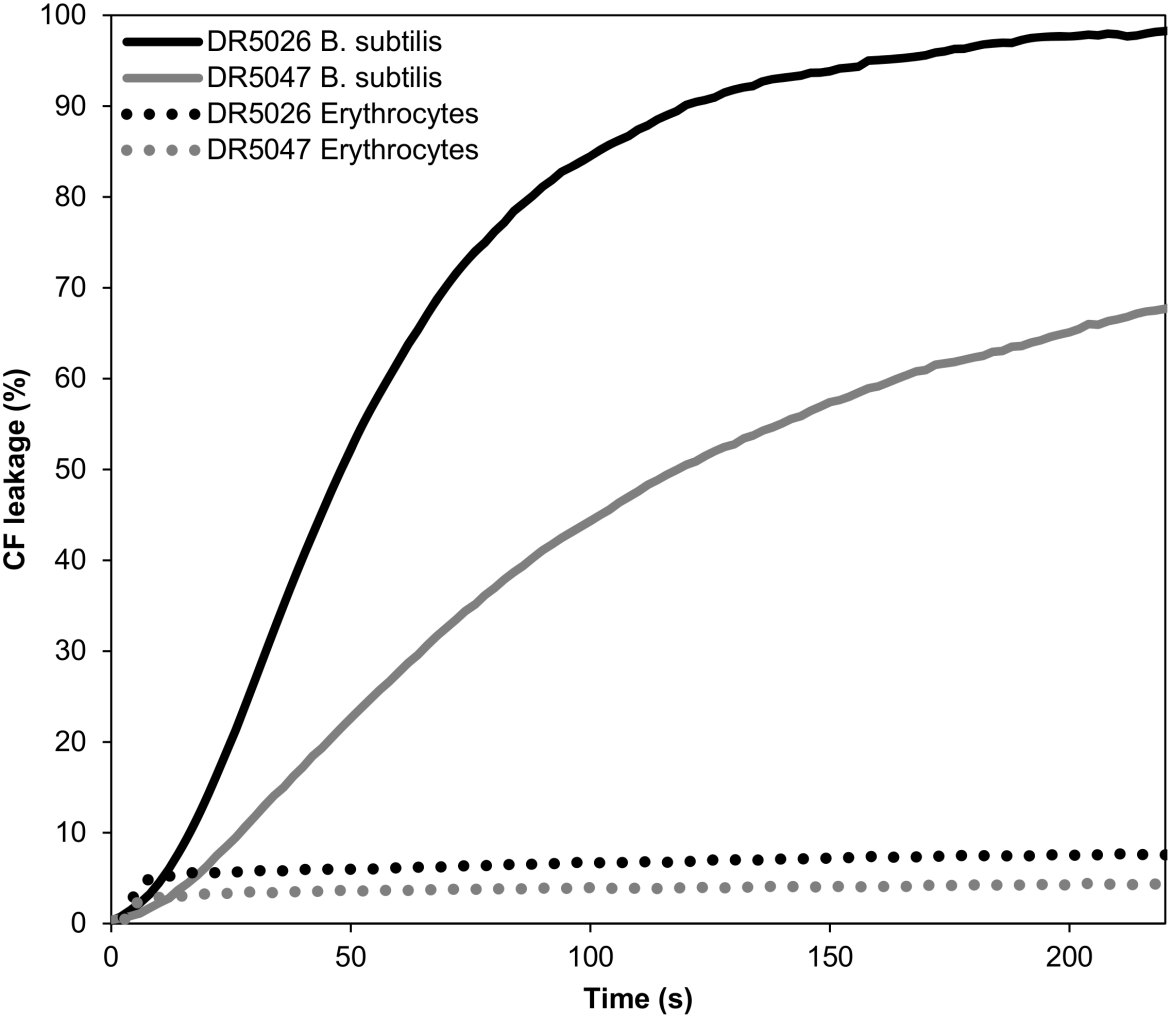
Leakage of contents for LUVs of different lipid composition measured by the release of CF (increase in fluorescence intensity). The maximum CF release was induced by 0.1% (v/v) Triton X-100. Total phospholipid concentration was 60 μM and temperature was kept at 25°C. LPPO concentration was 0.8 mg/L.

Next, we assessed the ability of LPPOs to disrupt lipid membranes using liposome leakage assay employing a suspensions of large unilamellar vesicles (LUV) loaded with 5(6)-carboxyfluorescein (CF) releasing its contents after the addition of LPPOs, which was recorded as an increase of sample fluorescence. The liposomes were prepared either from *B*. *subtilis* or eukaryotic (human erythrocytes) plasma membrane lipids. Both LPPOs efficiently disrupted the bacterial membrane leading to the loss of the internal vesicle contents (Fig 5). The leakage was faster and more efficient in the case of **DR5026** than **DR5047**. For both LPPOs the susceptibility to permeabilization dramatically differed between vesicles prepared from bacterial and eukaryotic lipids, the latter being substantially more resistant to lysis. Leakage of liposomes prepared from the bacterial phospholipids exhibited a biphasic behavior. CF was released in a rapid initial phase characterized by Hill coefficient *n* < 1 (n = 0.5 ± 0.2 and n = 0.7 ± 0.1 for **DR5026** and **DR5047**, respectively) followed by a delayed phase. In this latter phase the Hill coefficient was *n* = 4 ±1 for both LPPOs (S3 Fig) indicating a positive cooperativity of the LPPO molecules, i.e. that probably at least four molecules were required to possibly form a pore in the vesicle. In the case of LPPO-induced membrane permeabilization of vesicles prepared from erythrocyte lipids, LPPOs showed much lower efficacy (only 8% and 4% of the maximum CF release for **DR5026** and **DR5047**, respectively) and non-cooperative behavior (*n* < 1).

### LPPOs make pores in planar phospholipid bilayer

**Fig 6:**
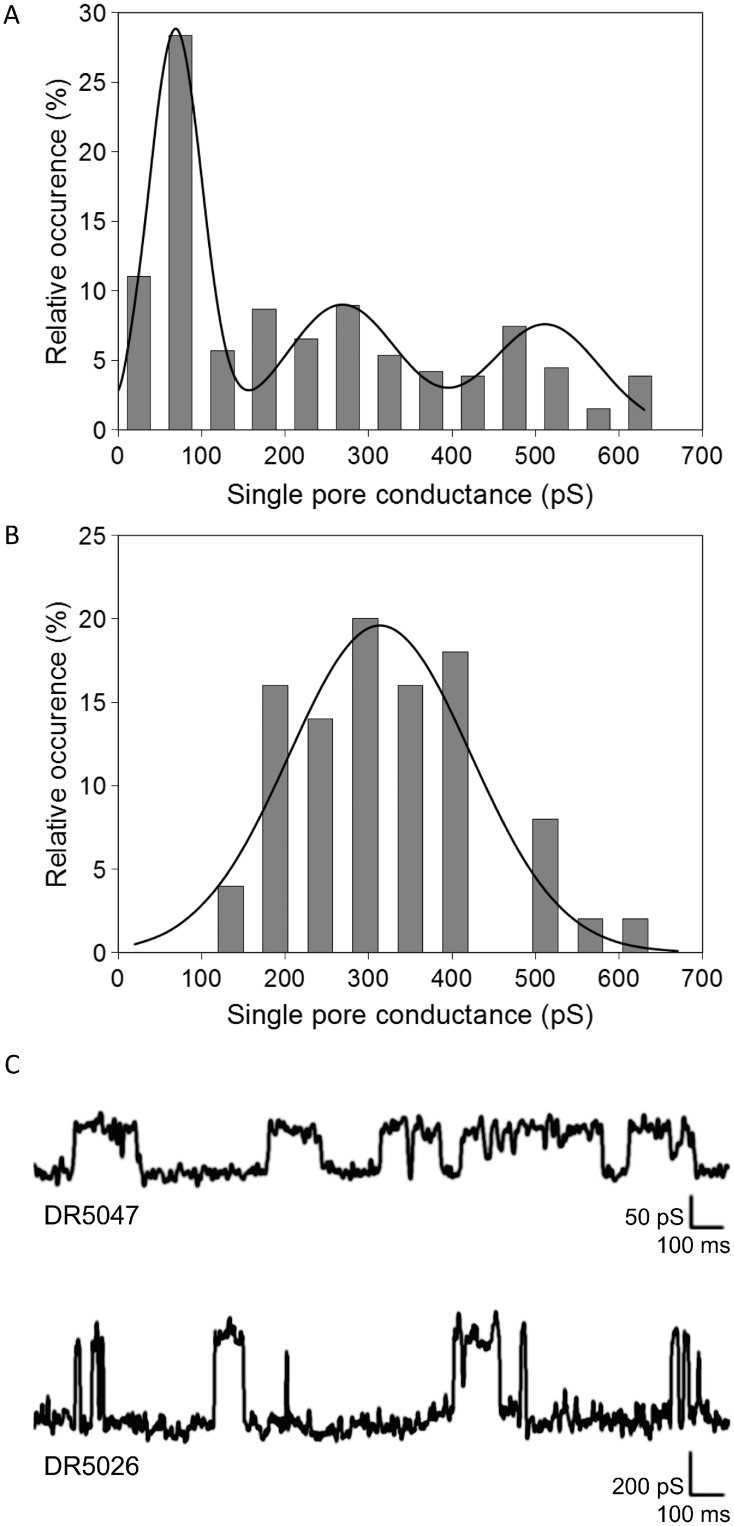
Conductance. Conductance of **DR5047** (**A**) and **DR5026** (**B**) single pores measured in 1M KCl, 10 mM Tris, pH 7.4 at membrane potential of 45 mV. The histograms of different conductance states were fitted with Gaussian functions. **C**. Representative single channel recordings of **DR5047** and **DR5026** in planar lipid membranes.

To investigate in detail the permeabilizing effect of LPPOs we tested them for pore-forming activity by conductance measurements in planar lipid membranes (black lipid membranes, BLM) formed from asolectin. When LPPOs were added, abrupt stepwise variations in membrane current were observed, indicative of opening and closing of individual pores. The pore conductance histograms and the representative current traces are shown in Fig 6. Consistent with the faster CF leakage rate from liposomes induced by **DR5026**, this LPPO formed most frequently pores of higher conductance than **DR5047**. The most frequent single pore conductance step observed for **DR5026** was 310 ± 128 pS. The variance of pore conductance was rather broad for **DR5047**, and could be described by three conductance distributions. The most frequent conductance step resulted in 70 ± 37 pS; however, conductance states of 270 ± 79 pS and 510 ± 77 pS were observed as well. The sizes of pores formed by both LPPOs are within the range of other well-characterized pore-forming agents. Under comparable experimental conditions (1 M salt solution) the LPPOs’ major conductance units were larger than those induced by gramicidin A that forms ca. 20 pS channels, which are ~0.4 nm wide [42, 43]. On the other hand, a more potent pore-former such as alamethicin forms ca. 600 pS channels of ~2 nm in diameter.

### Membrane permeabilization by LPPOs of *B*. *subtilis*, *S*. *epidermidis*, *E*. *faecalis*, *and* mouse macrophages

**Fig 7:**
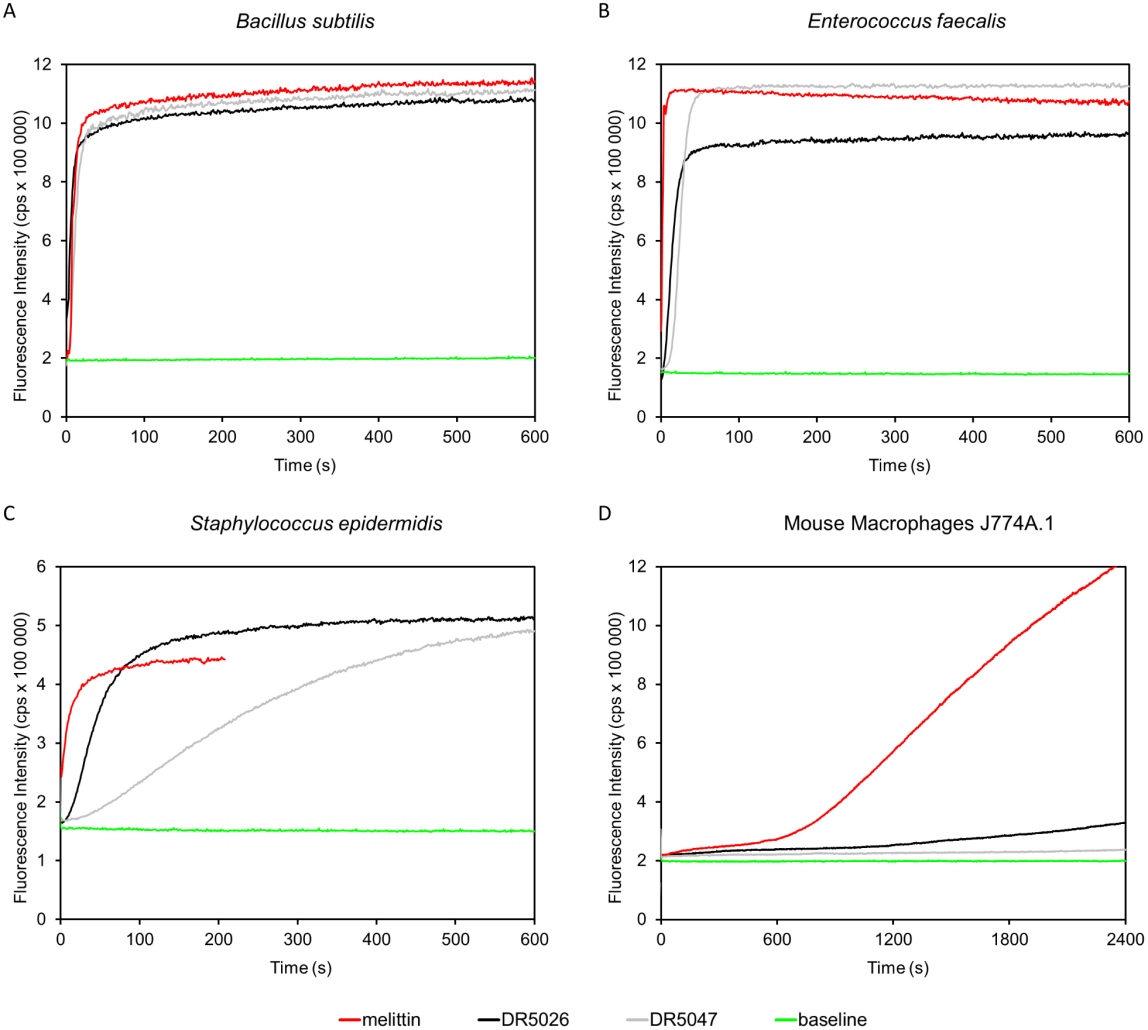
Permeabilization of cytoplasmic membrane induced by DR5026 and DR5047. At time 0 s permeabilizing agents were added to the cells at final concentrations of 10 mg/L or 10 μM for LPPOs and melittin, respectively. Increase in fluorescence intensity signifies that the membrane impermeant dye PI entered the cells.

To directly study the permeabilizing activity of LPPOs on live cells we determined their effect on membrane permeability by measuring the influx of propidium iodide (PI) to the bacterial cells. The dye enters only cells with compromised membrane, intercalates into double stranded DNA or RNA, resulting in fluorescence increase. The addition of either tested LPPO at 10 μg/ml induced rapid uptake of PI to *B*. *subtilis*, *E*. *faecalis* and *S*. *epidermidis* cells within several seconds, indicating fast kinetics (Fig 7). Lowering the concentration slowed down the kinetics but the compound remained active (S4 Fig). Finally and importantly, neither LPPO affected the cytoplasmic membrane permeability of mouse macrophages while a known pore former melittin [44] did so.

### Mode of interaction of LPPOs with the membrane

**Fig 8:**
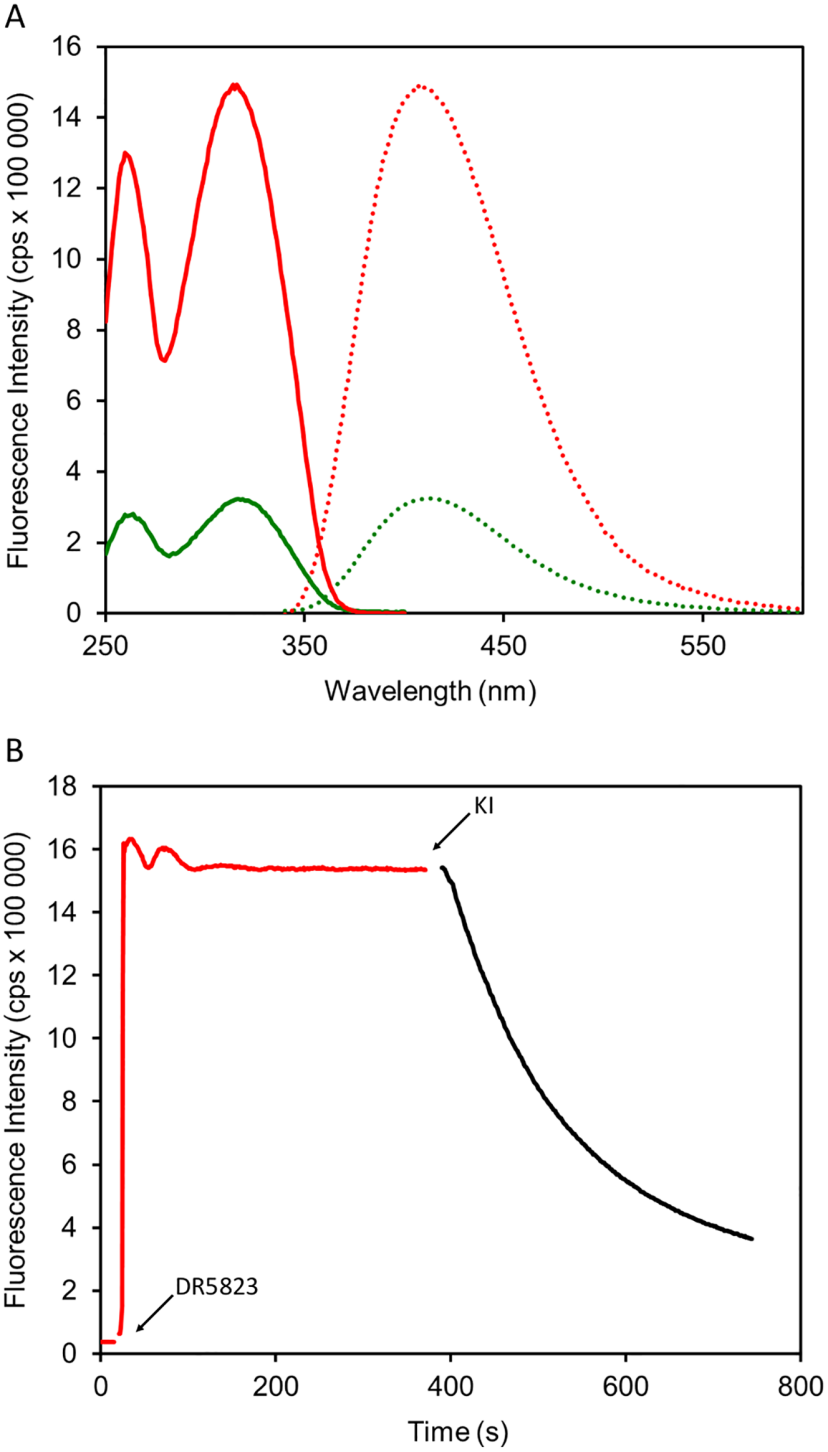
Interaction of fluorescently labeled LPPO DR5823 with model membrane. (A) Excitation (solid line) and emission (dotted line) spectra of **DR5823** in buffer (in green) and liposomes (in red). (B) Incorporation of **DR5823** to the liposome membrane.

To provide insights into the mechanism of LPPO interaction with the membrane we designed and synthesized LPPO **DR5823** –a fluorescently labeled analog of **DR5026** with minimal structural changes to avoid alteration of its physicochemical and biological properties (the structure, synthesis, and antibacterial activity of **DR5823** are described in S1 Supporting Information and S1 Table respectively). As model membrane we used dioleoylphosphatidylcholine (DOPC) bilayers. Upon **DR5823** addition fluorescence intensity substantially increased (4.5 times in comparison to buffer) (Fig 8A), suggesting LPPO incorporation into the non-polar environment of the membrane. Excitation spectrum showed maxima at 261 and 315 nm and fluorescence emission dominated at 411 nm.

Next, we tested the kinetics of the binding of **DR5823** to the membrane (Fig 8B). After addition of **DR5823** we observed a rapid increase of fluorescence intensity followed by multiphasic kinetics. Similar behavior could be observed with other membrane binding fluorescent probes such as 1,6-diphenyl-1,3,5-hexatriene (not shown) where these effects were explained by several phases of probe absorption and subsequent penetration into the membrane interior [45]. We further tested the accessibility of the **DR5823** fluorescent moiety to externally added quencher KI. Surprisingly, we recorded slow decrease in fluorescence intensity, suggesting gradual influx of KI into liposomes with **DR5823** incorporated possibly in both membrane leaflets.

**Fig 9:**
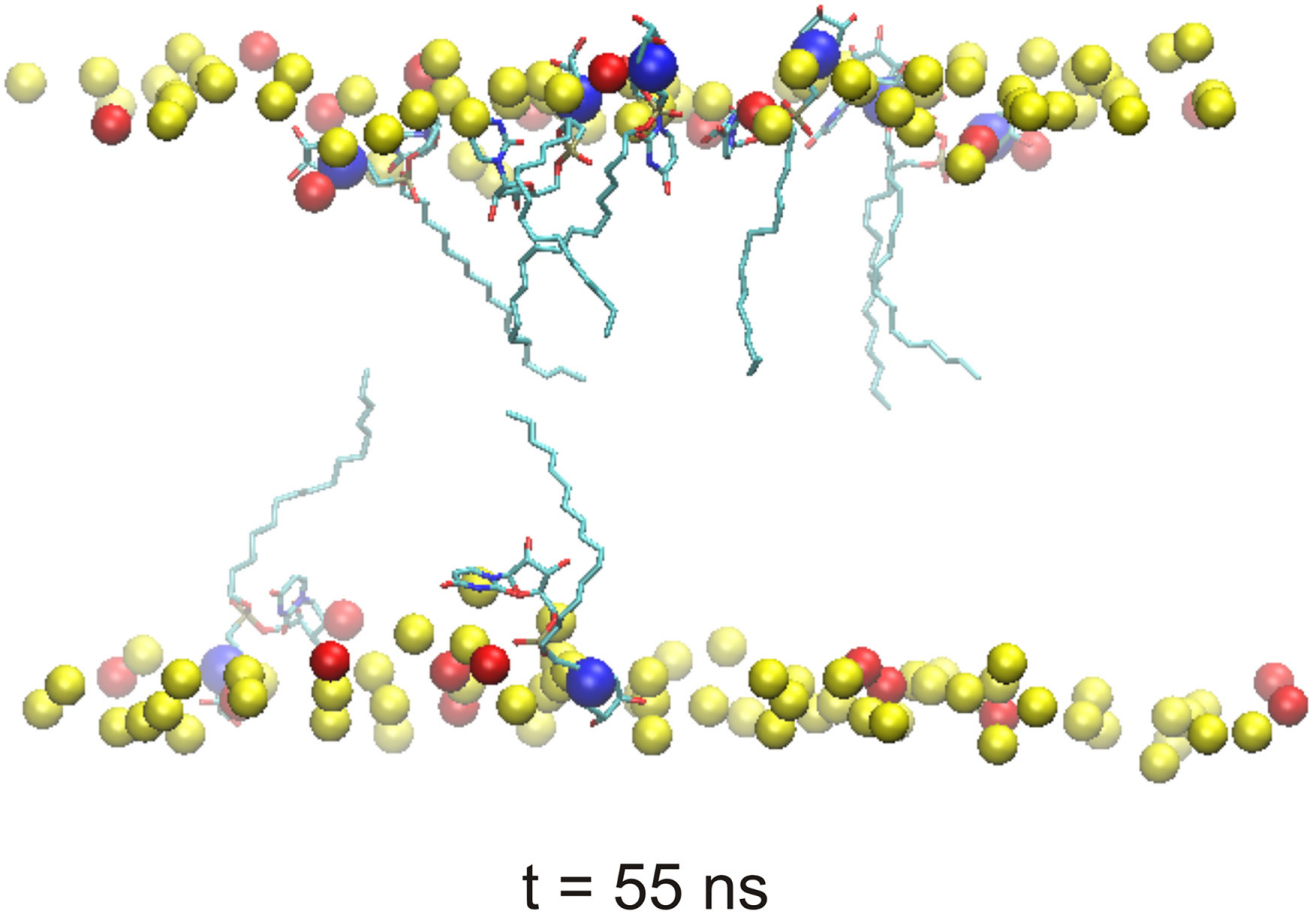
A model of the interaction of DR5026 with a bacterial membrane. The final state of MD simulation after 55 ns shows **DR5026** molecules penetrated into the phospholipid bilayer. The nitrogen atoms from the iminosugar modules of **DR5026** are highlighted as blue spheres. Phosphorus atoms of the phospholipid bilayer (PB) are depicted as yellow/red spheres. For clarity, almost all atoms of the PB are hidden.

These results were in agreement with *in silico* molecular dynamic (MD) simulations that suggested that LPPOs may function by forming pores in the membrane by being present in both membrane layers (Fig 9; and Figure A and Figure B in S2 Supporting Information; for more details see S2 Supporting Information). In the MD simulations the high affinity of **DR5026** for the model membrane seemed to arise from electrostatic interactions between the cationic iminosugar modules of LPPOs and the head groups of anionic lipids. Moreover, the hydrophobicity of lipophilic alkyl chains of LPPOs as well as the hydrophobicity of parts of uracil bases from nucleoside modules of LPPOs drove **DR5026** (with the exception of their iminosugar modules) deeper into the lipid tail region of the membrane. It resulted in an amphiphilic positioning of **DR5026** with the cationic iminosugar modules located at the lipid-water interface where they were anchored towards the membrane anionic head groups.

### Physicochemical properties and stability of LPPOs

Before proceeding to genotoxicity tests and minimum tolerated dose (MTD) experiments, we characterized LPPOs with respect to their physicochemical properties and stability. Both **DR5047** and **DR5026** were well soluble in water (>10 mg/ml). The CMC in H_2_O (measured using conductometry [35]) at 37°C were 3 mM (2 g/L) and 4 mM (3 g/L) respectively. LogD values that reflect lipophilicity of tested compounds were calculated using ACD/Labs software [36] and are shown in Table 2 (See also S4 Supporting Information).

**Table 2:** Calculated LogD values for DR-5047 and DR-5026.

DR-5047		DR-5026	
pH	LogD	pH	LogD
1.7	0.73	1.7	0.61
4.6	1.03	4.6	0.91
6.5	2.62	6.5	2.48
7.4	3.35	7.4	3.12
8.0	3.63	8.0	3.51

We assessed the stability of **DR5026** at three pH values (0.18, 5.00, and 8.80). The integrity of the sample was tested at two concentrations (50 μM and 500 μM) and two temperatures (at room temperature and at 37°C), and it was measured using LC-MS after 24 hours of incubation. At both sample concentrations and regardless of the temperature, the LPPO displayed excellent stability at all the tested pH values with no detectable decomposition.

### Genotoxicity

Previously, selected LPPOs were tested for their cytotoxicity against human cell lines, displaying cytotoxic concentrations well above their MIC against bacteria [21]. Here, we tested the mutagenic potential **DR5026** and **DR5047** by the standard Ames test. Neither of the two compounds was found to be genotoxic in these tests at relevant concentrations.

### Maximum Tolerated Dose (MTD) for DR5026 in mice

To establish the tolerance for LPPOs in mammals, we selected **DR5026** and mice as our model system. For each dose, 3 males and 3 females were tested.

#### Administration per os

The doses of 5, 500, 1000, 1500 and 2000 mg/kg of body weight did not cause any signs of toxicity. Moreover, no gross pathology changes were detected (for details see S3 Supporting Information).

#### Administration intraperitoneally

Doses of 5, 50, 80, 250, and 500 mg/kg of body weight were tested. The doses of 5 and 50 mg/kg of body weight did not cause any signs of toxicity. After the administration of 80 mg/kg all the monitored animals were slightly stolid 5 min after the administration. This sign disappeared in 2 males and 3 females within 6 hours after the administration. In one male a disturbance of locomotion was observed 24 hours after the administration; this moribund animal was sacrificed 26 hours after the administration. Increasing the dose to 250 mg/kg and 500 mg/kg, respectively, exacerbated the symptoms; administration of the higher dose lead to death of 2 males and 2 females several hours after the administration (for Details see S3 Supporting Information).

In conclusion, **DR5026** displayed promising maximal tolerated dose parameters for peroral administration when even the highest dose (2000 mg/kg) did not reveal any signs of toxicity. When administered peritoneally, the safe limit was 50 mg/kg, and this suggested that LPPOs may not be suitable for systemic applications but they may offer a good alternative for topical and/or gastrointestinal applications.

### Transepithelial transport of LPPO in Caco-2 monolayers

To investigate the potential of LPPOs for the treatment of gastrointestinal infections, **DR-5026** was tested for transepithelial transport in a Caco-2 monolayer. The tested compound was detected at the apical (donor) side at the expected concentration (200 μM). No compound was detected at the basolateral (acceptor) side as well as in the Caco-2 cell lysate, indicating that the tested LPPO neither passed through the Caco-2 monolayer nor was absorbed by the cells.

### The potential for resistance against LPPOs

**Fig 10:**
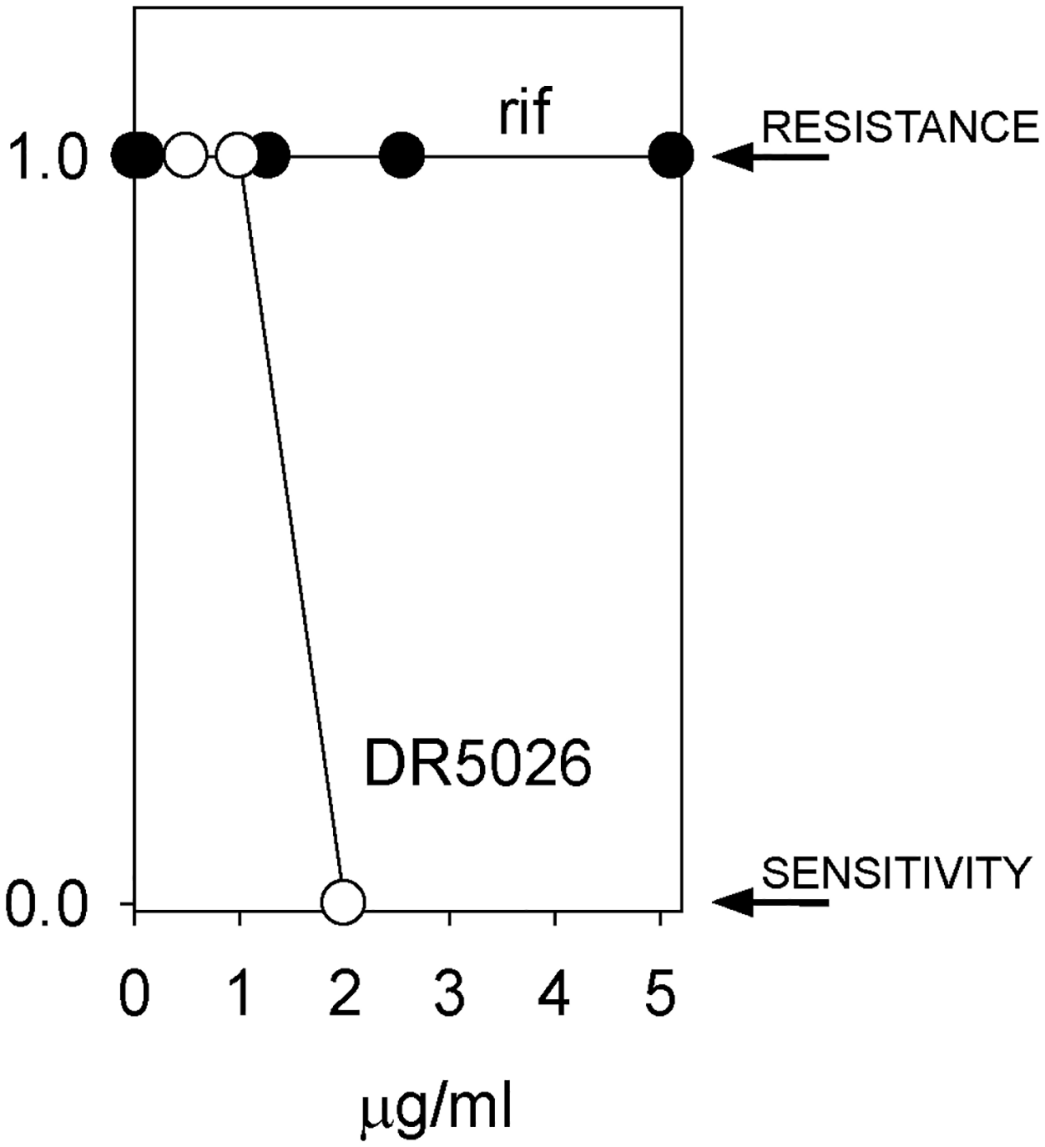
*B*. *subtilis* 168 develops resistance against rifampicin (rif) but not against DR5026. *B*. *subtilis* was incubated with subcytotoxic concentrations of rif (starting at 0.01 mg/L; MIC 0.06 mg/L) and **DR5026** (0.5 mg/L MIC ~ 3 mg/L) and grown for 24 h. Then, aliquots of the cultures were transferred to new tubes with fresh medium and a two-fold increased concentration of the active compound. A binary representation is shown; 1 indicates growth of the cells, i. e. their resistance to the respective compound; 0 represents lack of growth—the cells were sensitive to the compound and no resistant cell appeared within the time frame of the experiment. The experiment was conducted in three biological replicates with the same results.

Finally we tested the potential for the development of resistance against LPPOs in *B*. *subtilis*. Rifampicin was used as a control. We started culturing the cells in the presence of subcytotoxic concentrations of either **DR5026** or rifampicin. The cells were incubated for 24 hours after which they were diluted into fresh medium containing a two-fold increased concentration of the respective compound. This scheme was followed for several days. Fig 10 shows that while we were able to obtain cells growing in the presence of 5 mg/L of rifampicin (MIC against wt *B*. *subtilis* ~0.06 mg/L), we failed to obtain cells that would grow at concentrations exceeding the MIC of **DR5026** (~3 mg/L). Subsequently, the induction of resistance of clinically more relevant species was carried out by repeated passages of *E*. *faecalis* and *S*. *agalactiae* strains with subinhibitory concentrations of LPPOs. A total of 14 passages were performed, after each the MICs were determined. Similarly to *B*. *subtilis*, the MICs remained relatively unchanged (S2 Table).

## Discussion

In this study, we described the mechanism of action of novel antibacterial compounds, lipophosphonoxins. We showed that LPPOs did not inhibit biosynthesis of main macromolecules (DNA, RNA, proteins, lipids, cell wall peptidoglycan; Fig 2). However, transmission electron microscopy suggested that LPPOs caused rapid damage to the bacterial cell envelope (Fig 3), resulting in leakage of internal content and, ultimately, complete cell lysis. Subsequent experiments demonstrated that LPPOs did indeed accumulate in bacterial cellular membranes (Fig 4). Finally, the membrane damage and its mechanism were addressed by CF leakage, BLM, PI-penetration, and fluorescent LPPO-membrane interaction experiments that revealed that LPPOs likely disrupted the membrane by forming pores in it, possibly by being present on both sides of the phospholipid bilayer (Figs 5–8), The pore formation was specific for bacterial membranes whereas their effect on membranes derived from human erythrocytes and macrophages was at least an order of magnitude less pronounced (Figs 5 and 7). No protein was required for the action of LPPOs. This correlated with our inability to find a protein binding partner of LPPOs (S1 Fig), although we note that by this approach we would be able to detect only proteins with strong affinity for LPPOs. The pore formation was also corroborated by MD simulations that suggested that LPPOs may cause local transient changes in membrane curvature (Fig 9; Figure A and Figure B in S2 Supporting Information). Such changes may also compromise the membrane integrity and lead to its disruption. Importantly, strains resistant against membrane active compounds are difficult/slow to emerge and this also seemed to be the case with LPPOs (Fig 10).

We used non-pathogenic *B*. *subtilis* as our model organism for experiments throughout the study. Nevertheless, PI-penetration, and development of resistance experiments demonstrated that the results obtained with this bacterium are valid also for clinically relevant species (*E*. *faecalis*, *S*. *epidermidis*). Still, we note that LPPOs display differential effect against different bacterial species. This is possibly caused by species-to-species differences in the phospholipid composition of the plasmatic membrane, which was also shown for membrane targeting antibiotics such as gramicidin [46] or daptomycin [47]. Experiments are in progress, addressing the membrane composition determinants that make LPPOs selective against bacterial membranes.

LPPOs belong to the growing family of small molecule antibacterial peptide mimetics, that include cationic steroid antibiotics [48–50], phenylalanine lipophilic norspermidine derivatives [51], xanthone-based, membrane-targeting antimicrobials [52], 2-((3-(3,6-dichloro-9H-carbazol-9-yl)-2-hydroxypropyl)amino)-2-(hydroxymethyl)propane-1,3-diol (DCAP) [53], arylamide foldamers [54, 55], and a promising synthetic, bactericidal antimicrobial peptide, which degrades the membranes of harmful micro-organisms coded as LTX-109 [56]. These compounds are structurally heterogenic but they all are amphiphilic molecules containing a lipophilic part and hydrophilic part containing usually positive charge(s). LPPOs also share this structural feature, however, a major advantage of LPPOs is their modular structure allowing for systematical structure activity relationship study. Such studies are, in fact, currently in progress and should result in improved properties of these compounds, e. g. increasing their selectivity and extending their antibacterial activity also to serious Gram-negative pathogens such as *Pseudomonas aeruginosa*.

The lack of genotoxicity of LPPOs is not surprising as they do not enter the cytosol. As reported in our previous study, they were not cytotoxic against human cell lines at their bactericidal concentrations [21], and here we have shown that they were well tolerated in mammals when administered orally. However, the adverse effects of the higher dose when used peritoneally strongly argue against their use as systemic antibiotics. Nevertheless, there is a need for topical antibiotics dictated by an increasing number of superficial skin infections caused by multiresistant bacteria such as *Streptococcus pyogenes* with constitutive or inducible macrolide-lincosamide-streptogramin (MLS) phenotype of resistance or vancomycin-resistant enterococci [57]. An example of a compound being developed for topical treatment of skin infections and nasal eradication of *staphylococci* is LTX-109. It has already passed in man Phases 1, 1/2, and 2a (Studies C08-109-01; C10-109-02). Extending the portfolio of such compounds is highly desirable.

The lack of toxicity of LPPOs when administered perorally, their stability at a wide range of pH, and their inability to be absorbed in the intestine suggest that they could be developed also into antibiotics for the treatment of GIT infections such as the commonly occurring coinfection by vancomycin-resistant *Enterococcus* and *Clostridium difficile* [58]. Applications in the prophylaxis and treatment of infections in joint surgery, where LPPO sensitive methicillin-resistant *S*. *epidermidis* 8700/B (**DR-5026** MIC = 3.125 mg/L) often plays an important role, are other areas of potential use. Experiments on infection models will be required to address their ability to eradicate pathogens in living organisms, and results of these experiments will be reported in due course.

## Ethics Statement

### Facilities management and animal husbandry

Animal care was in compliance with the SOPs of MediTox s.r.o., the European convention for the protection of vertebrate animals used for experimental and other scientific purposes (ETS 123), the Czech Collection of laws No. 246/1992, inclusive of the amendments, on the Protection of animals against cruelty, and Public Notice of the Ministry of Agriculture of the Czech Republic, Collection of laws No. 419/2012 as amended, on keeping and exploitation of experimental animals. MediTox s.r.o., is a holder of the accreditation Certificate for user’s issued by Central Committee for Animal Protection of the Czech Republic.

### Animal welfare act compliance

The study was prepared for this type of experiment and approved by the Institutional Animal Care and Use Committee (IACUC) and the Committee for Animal Protection of the Ministry of Health of the Czech Republic (PP 09/2014). Procedures used in this plan were designed to conform to accepted practices and to minimize or avoid causing pain, distress, or discomfort to the animals. In those circumstances in which required study procedures were likely to cause more than momentary or slight pain or distress, the animals received appropriate analgesics or anesthetics unless the withholding of these agents had been justified in writing by the Study Director and/or the Sponsor and approved by the Institutional Animal Care and Use Committee (IACUC) at MediTox s.r.o. The number of animals selected for use in this study was considered to be the minimum number necessary to meet scientific and regulatory guidelines for this type of study.

## Supporting Information

S1 Fig Search for a potential protein binding partner(s) of LPPOs *B*. *subtilis* cell lysate was incubated either with empty streptavidin beads (EB) or with the beads couples with biotinylated LPPO (DR5690). The proteins associating with EB and LPPO were then resolved on SDS—PAGE and stained with Commassie Blue. A labeled molecular weight ladder (M) is shown on the left. The experiment was conducted three times on different days. A representative experiment is shown.
(PDF)

S2 Fig Structures of potential metabolites of DR5047. In all HPLC-MS analyses only intact **DR5026** was detected. In the case of **DR5047** (blue rectangle) less than 5% of the compound with the exact mass of 761.42 was detected. It corresponds to a monoacetylated compound—highlighted by the green rectangle—the exact position of the acetyl group has not been determined (other possible positions for the acetyl group are indicated with red arrows).
(PDF)

S3 Fig CF release curve fitting. Time course of liposome lysis induced by **DR5026** (A). Solid lines show the fit of the functions: *α*(1-exp(-*t*/τ))^*n*^ to the data. Leakage of liposomes prepared from the bacterial phospholipids exhibited a biphasic behavior—in this data set the parameters for the fit of the initial phase (f(x)) were *α* = 29.7, τ = 175.5 s, *n* = 0.6 and for the latter phase (g(x)) were *α* = 76.4, τ = 28.2 s, *n* = 3.1. The difference between the experimental data and the model (sum f(x) and g(x)) is shown in (B).
(PDF)

S4 Fig Concentration-dependent cytoplasmic membrane permeabilization induced by DR5026. The increase in fluorescence intensity reflected intercalation of PI into DNA and dsRNA that occurred after PI entry into the cell upon membrane permeabilization.
(PDF)

S1 Supporting Information Synthesis of DR5557, biotinylated LPPO DR5690, and fluorescently labeled LPPO DR5832. (PDF)

S2 Supporting Information MD simulations. (PDF)

S3 Supporting Information MTD test in mouse model—a detailed description. (PDF)

S4 Supporting Information LogD calculation. (PDF)

S1 Table Antibacterial activity of LPPOs DR5557, DR5690, and DR5823. (PDF)

S2 Table The induction of resistance of *Enterococcus faecalis* and *Streptococcus agalactiae* to compounds DR5047 and DR5026. Minimum inhibitory concentrations of the tested LPPOs after each induction step. The induction of resistance was carried out by repeated passages of *E*. *faecalis* and *S*. *agalactiae* strains with subinhibitory concentrations of the relevant LPPOs. A total of 14 passages were performed. The results clearly showed that the MICs remained relatively unchanged. It meant that the resistance was not induced.
(PDF)
