# Supplementary material for: Insights into the Mechanism of Action of Bactericidal Lipophosphonoxins
Source: PLoS One. 2015 Dec 30;10(12):e0145918. doi: 10.1371/journal.pone.0145918 (PMC4696656; doi:10.1371/journal.pone.0145918)
Supplement: S1 Fig — The proteins associating with EB and LPPO were then resolved on SDS—PAGE and stained with Commassie Blue. A labeled molecular weight ladder (M) is shown on the left. The experiment was conducted three times on different days. A representative experiment is shown. (PDF) [file pone.0145918.s001.pdf]

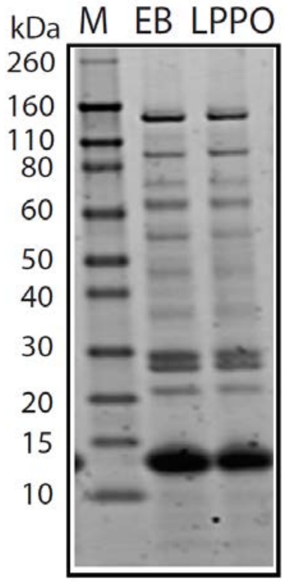

S1 Fig. Search for a potential protein binding partner(s) of LPPOs *B. subtilis* cell lysate was incubated either with empty streptavidin beads (EB) or with the beads couples with biotinylated LPPO (**DR5690**). The proteins associating with EB and LPPO were then resolved on SDS – PAGE and stained with Commassie Blue. A labeled molecular weight ladder (M) is shown on the left. The experiment was conducted three times on different days. A representative experiment is shown.
