# Supplementary material for: Insights into the Mechanism of Action of Bactericidal Lipophosphonoxins
Source: PLoS One. 2015 Dec 30;10(12):e0145918. doi: 10.1371/journal.pone.0145918 (PMC4696656; doi:10.1371/journal.pone.0145918)
Supplement: S2 Fig — In all HPLC-MS analyses only intact DR5026 was detected. In the case of DR5047 (blue rectangle) less than 5% of the compound with the exact mass of 761.42 was detected. It corresponds to a monoacetylated compound—highlighted by the green rectangle—the exact position of the acetyl group has not been determined (other possible positions for the acetyl group are indicated with red arrows). (PDF) [file pone.0145918.s002.pdf]

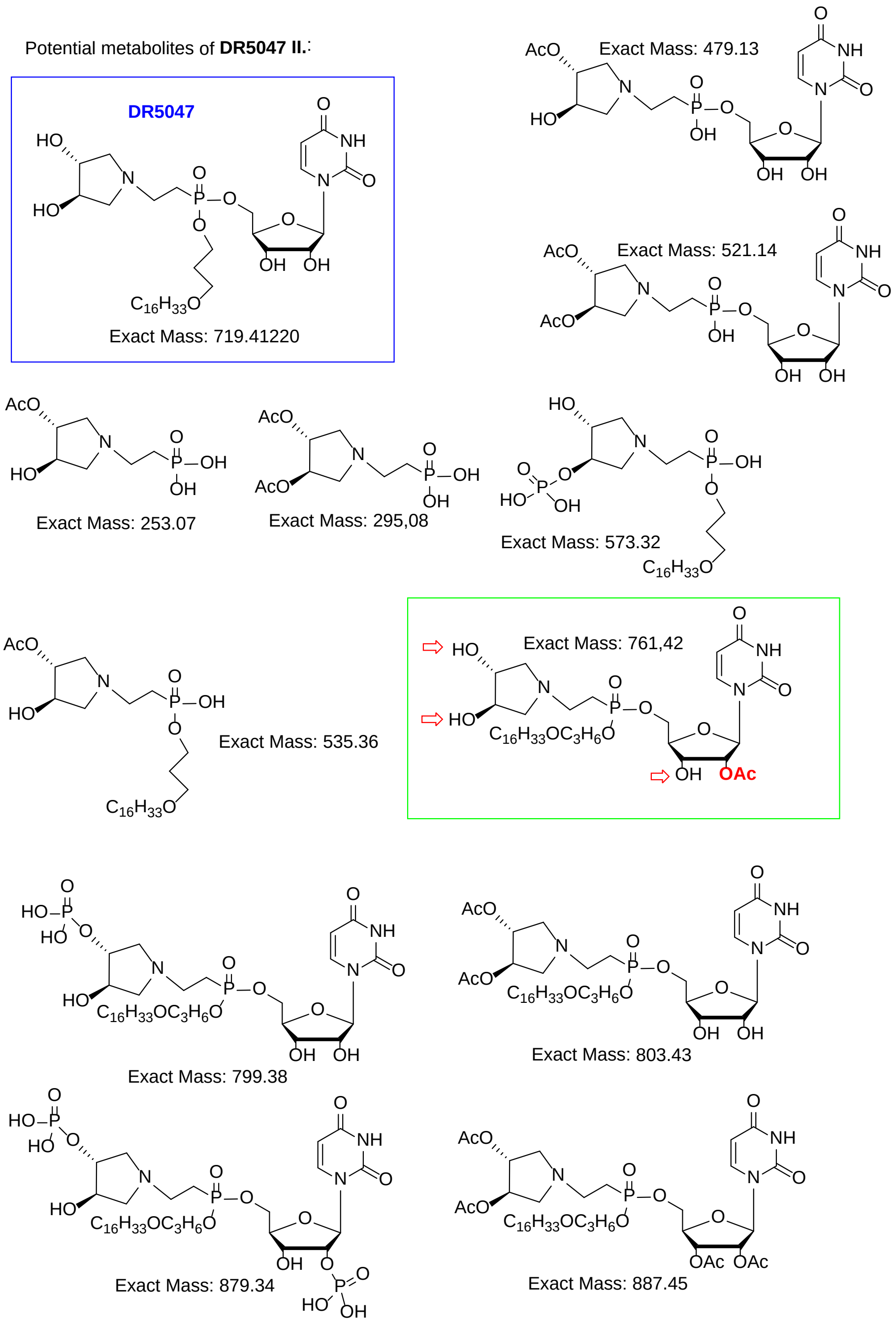

S2 Fig. Structures of potential metabolites of DR5047. In all HPLC-MS analyses only intact **DR5026** was detected. In the case of **DR5047** (blue rectangle) less than 5% of the compound with the exact mass of 761.42 was detected. It corresponds to a monoacetylated compound – highlighted by the green rectangle - the exact position of the acetyl group has not been determined (other possible positions for the acetyl group are indicated with red arrows).
