# Supplementary material for: Insights into the Mechanism of Action of Bactericidal Lipophosphonoxins
Source: PLoS One. 2015 Dec 30;10(12):e0145918. doi: 10.1371/journal.pone.0145918 (PMC4696656; doi:10.1371/journal.pone.0145918)
Supplement: S3 Fig — Time course of liposome lysis induced by DR5026 (A). Solid lines show the fit of the functions: α(1-exp(-t/τ))n to the data. Leakage of liposomes prepared from the bacterial phospholipids exhibited a biphasic behavior—in this data set the parameters for the fit of the initial phase (f(x)) were α = 29.7, τ = 175.5 s, n = 0.6 and for the latter phase (g(x)) were α = 76.4, τ = 28.2 s, n = 3.1. The difference between the experimental data and the model (sum f(x) and g(x)) is shown in (B). (PDF) [file pone.0145918.s003.pdf]

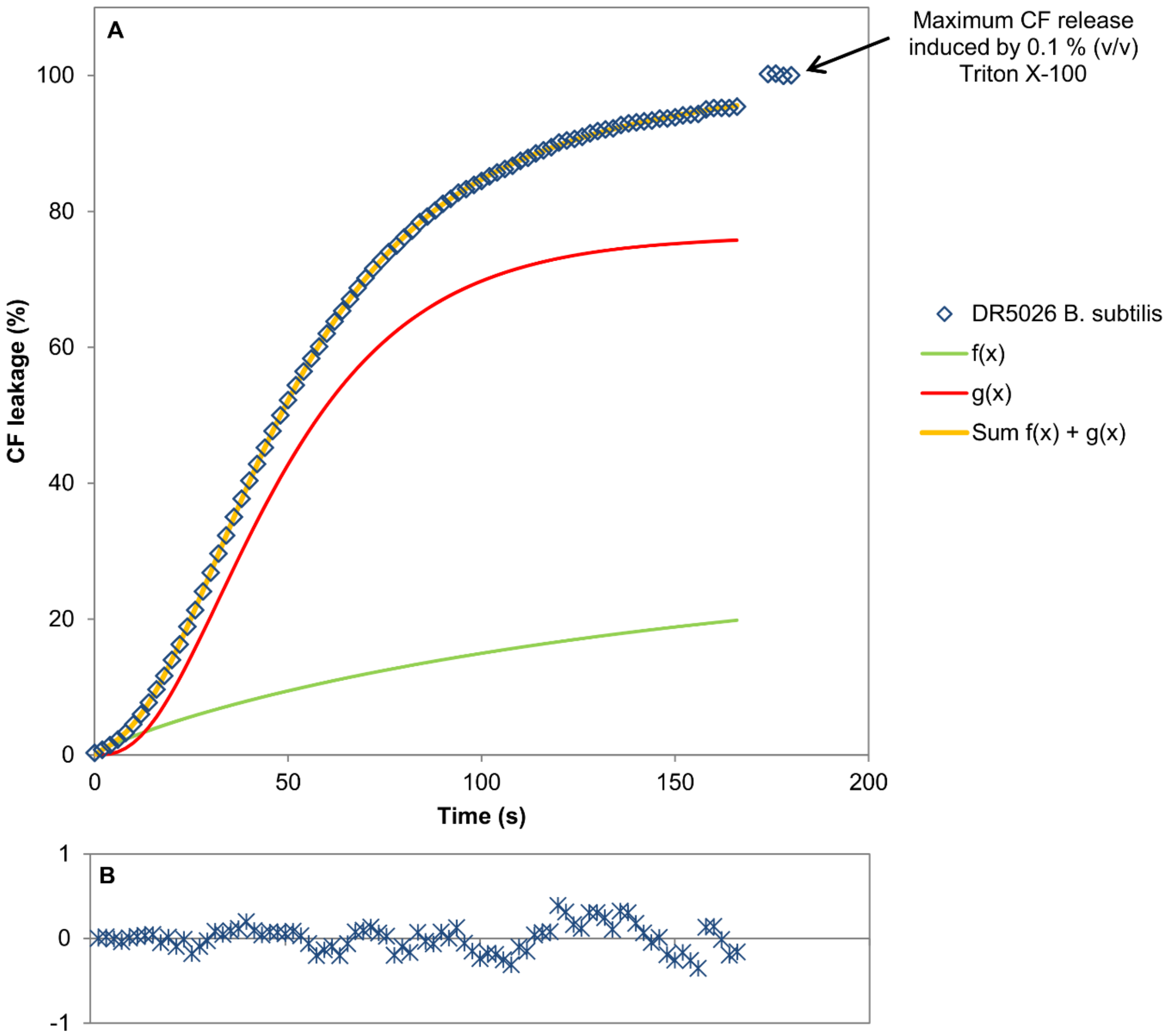

S3 Fig. CF release curve fitting. Time course of liposome lysis induced by **DR5026** (A). Solid lines show the fit of the functions: $\alpha(1\text{-exp}(-t/\tau))^n$ to the data. Leakage of liposomes prepared from the bacterial phospholipids exhibited a biphasic behavior – in this data set the parameters for the fit of the initial phase (f(x)) were α = 29.7, τ = 175.5 s, n = 0.6 and for the latter phase (g(x)) were α = 76.4, τ = 28.2 s, n = 3.1. The difference between the experimental data and the model (sum f(x) and g(x)) is shown in (B).
