# Supplementary material for: Insights into the Mechanism of Action of Bactericidal Lipophosphonoxins
Source: PLoS One. 2015 Dec 30;10(12):e0145918. doi: 10.1371/journal.pone.0145918 (PMC4696656; doi:10.1371/journal.pone.0145918)
Supplement: S4 Fig — The increase in fluorescence intensity reflected intercalation of PI into DNA and dsRNA that occurred after PI entry into the cell upon membrane permeabilization. (PDF) [file pone.0145918.s004.pdf]

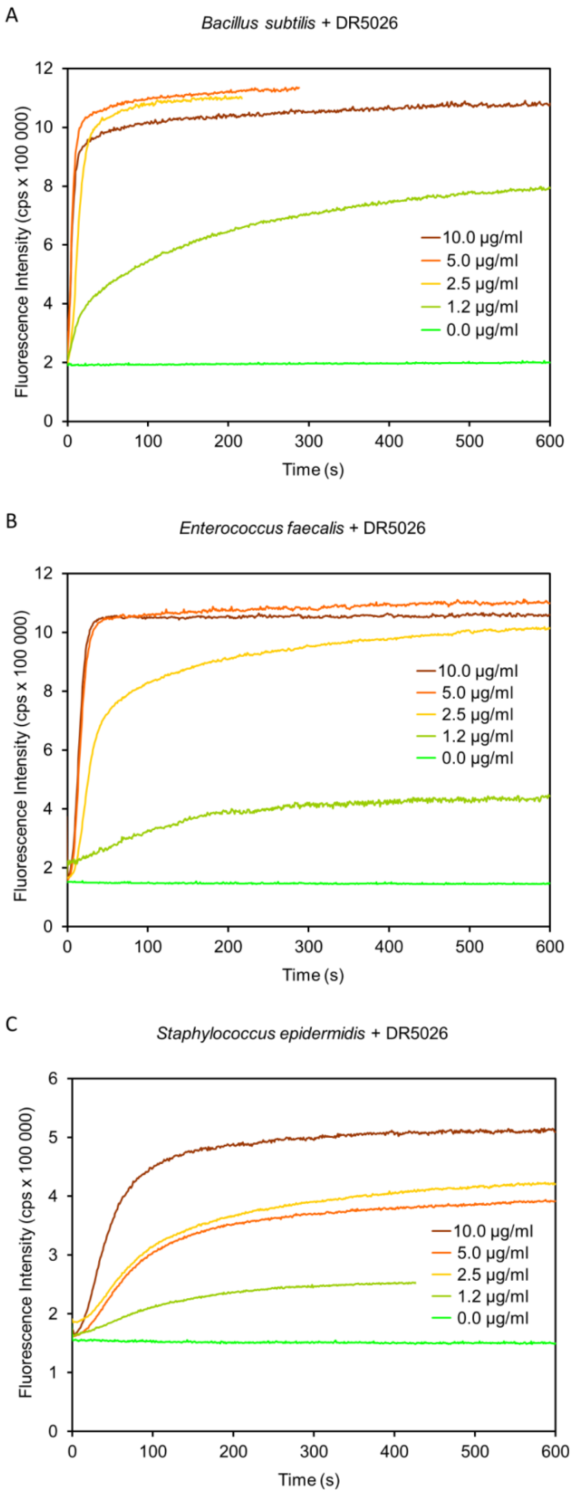

S4 Fig. Concentration-dependent cytoplasmic membrane permeabilization induced by DR5026. The increase in fluorescence intensity reflected intercalation of PI into DNA and dsRNA that occurred after PI entry into the cell upon membrane permeabilization.
