# Supplementary material for: Insights into the Mechanism of Action of Bactericidal Lipophosphonoxins
Source: PLoS One. 2015 Dec 30;10(12):e0145918. doi: 10.1371/journal.pone.0145918 (PMC4696656; doi:10.1371/journal.pone.0145918)
Supplement: S1 Supporting Information — (PDF) [file pone.0145918.s005.pdf]

Synthesis of DR5557, biotinylated LPPO DR5690, and fluorescently labeled LPPO DR5832

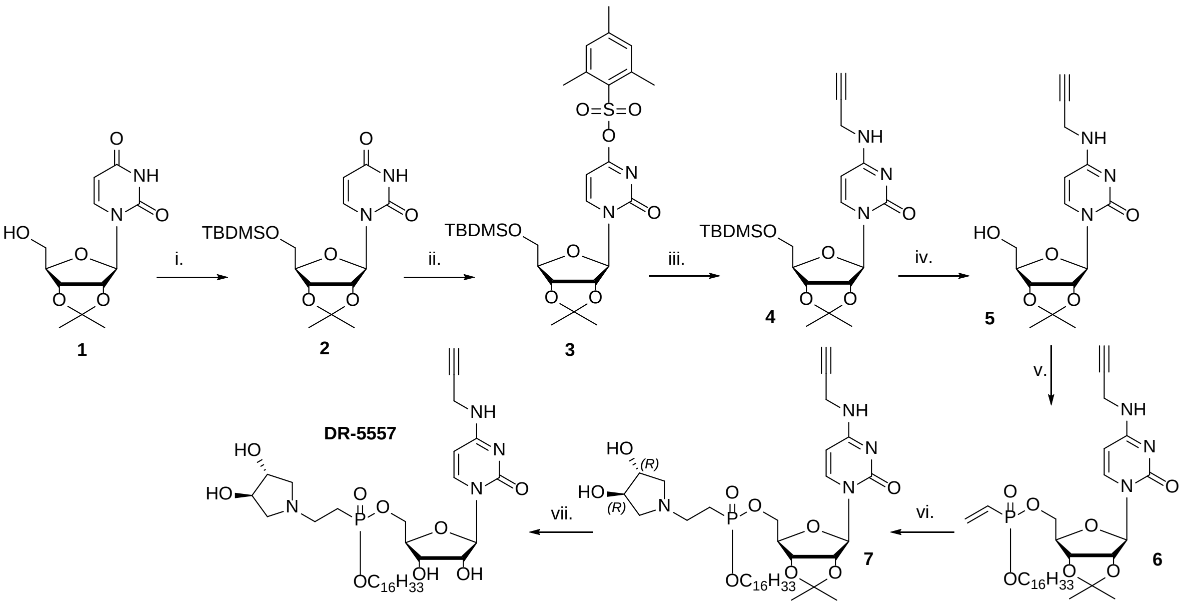

i.TBDMSCl, imidazol, pyridin; ii. MSCl, TEA, DMAP, DCM; iii. propargylamin, DMF; iv. Bu_4NF, THF; v.CH_2=CHP(O)(OR_3)(OH), TPSCl, DMAP, DCM; vi. dihydroxypyrrolidine, nBuOH; vii. 0.5M HCl/MeOH

Scheme A. Synthesis of compound DR5557.

General

Unless stated otherwise, all used solvents were anhydrous. TLC was performed on silica gel pre-coated aluminium plates Silica gel/TLC-cards, UV 254 (Fluka), and compounds were detected by UV light (254 nm), by heating (detection of dimethoxytrityl group; orange color), by spraying with 1% solution of ninhydrine to visualize amines, and by spraying with 1% solution of 4-(4-nitrobenzyl)pyridine in ethanol followed by heating and treating with gaseous ammonia (blue color of mono- and diesters of phosphonic acid). Preparative column chromatography was carried out on silica gel (40-60µm; Fluka), and elution was performed at the flow rate of 40 ml/min. The following solvent systems were used for TLC and preparative chromatography: toluene-ethyl acetate 1:1 (T); chloroform-ethanol 9:1 (C1); ethyl acetate-acetone-ethanol-water 6:1:1:0.5 (H3); ethyl acetate-acetone-ethanol-water 4:1:1:1 (H1). The concentrations of solvent systems are stated in volume percents (%, v/v). Purity of prepared compounds was determined by LC-MS performed on Waters AutoPurification System with 2545 Quarternary Gradient Module and 3100 Single Quadrupole Mass Detector using LUNA C18, column (Phenomenex, 100 x 4.6 mm, 3 µm) at flow rate 1 ml/min. Typical conditions: mobile phase, A - 50mM NH_4HCO_3; B - 50 mM NH_4HCO_3 in 50% aq. CH_3CN; C – CH_3CN; A→B/10 min, B→C/10 min, C/5 min. Preparative RP HPLC was performed on LC5000 Liquid Chromatograph (INGOS-PIKRON, CR) using Luna C18 (2) column (250 x 21.2 mm, 5 µm) at flow rate of 10 ml/min by a gradient elution of methanol in 0.1M TEAB pH 7.5 (A = 0.1M TEAB; B = 0.1M TEAB in 50% aq. methanol; C = methanol) or without buffer.

All final compounds were lyophilized from water. Mass spectra were recorded on LTQ Orbitrap XL (Thermo Fisher Scientific) using ESI ionization. NMR spectra were measured on Bruker AVANCE 400 (^{1}H at 400 MHz, ^{13}C at 100.6 MHz), Bruker AVANCE 500 and Varian UNITY 500 (^{1}H at 500 MHz, ^{13}C at 125.8 MHz) spectrometers. D_2O (reference (dioxane) = ^{1}H 3.75 ppm, ^{13}C 69.3 ppm. Chemical shifts (in ppm, δ scale) were referenced to TMS as internal standard; coupling constants (*J*) are given in Hz. All intermediates were determined by LC-MS.

Synthesis of DR5557

TBDMSCl (6.37 g, 42.27 mmol) was added to solution of 2',3'-isopropylideneuridine (**1**) (8.01 g, 28.18 mmol) in pyridine. The reaction mixture was stirred at rt overnight. Solvent was removed in vacuo and product **2** was obtained by chromatography on silica gel using linear gradient of ethyl acetate in toluene in 95% yield (10.7 g, 26.8 mmol). **2** was without further characterization dissolved in DCM (300 ml) and triethylamine (9 ml, 65 mmol), DMAP (0.2 g, 1.6 mmol) and mesitylenesulfonylchloride (14 g, 65 mmol) were added. The reaction mixture was stirred at rt for 3 h and washed with saturated solution of $NaHCO_3$. Organic phase was dried over Na_2SO_4, evaporated and intermediate **3** was obtained by chromatography on silica gel using linear gradient of ethyl acetate in toluene in 50% yield (7.8 g, 13.4 mmol). Intermediate **3** (2.78 g, 4.79 mmol) was (without further characterization) treated with propargylamine (0.61 ml, 9.57 mmol) in DMF (50 ml) at 90 °C overnight. The reaction mixture was concentrated in vacuo and treated with TBAF (0.5 M in THF, 30 ml) for 4 h. Dowex 50 in triethylammonium form (20 g) was added, the suspension was stirred for another 10 min, filtered and concentrated in vacuo. Intermediate **5** was obtained by linear gradient of ethanol in chloroform in 85% yield (1.31g, 4.05 mmol).
TPSCl (3.68 g, 12.15 mmol) was added to mixture of **5** (1.31 g, 4.05 mmol), hexadecyl vinylphosphonate (2.7 g, 8.1 mmol) and 1-methylimidazole (0.96 ml, 12.15 mmol) in DCM (80 ml). The reaction mixture was stirred at rt overnight, and washed with saturated solution of $NaHCO_3$. The vinylphosphonate intermediate **6** was obtained by linear gradient of ethanol in chloroform.
Mixture of vinylphosphonate **6** (0.32 g, 0.5 mmol) and [*R*,*R*]-3,4-dihydroxypyrrolidine (62 mg, 0.6 mmol) in nBuOH (5 ml) was stirred at 70 °C overnight. Solvent was removed in vacuo and the residue was treated with 0.5 M methanolic HCl (20 ml) overnight at rt. The reaction mixture was concentrated in vacuo and final **DR5557** was obtained by the chromatography on silica gel using linear gradient of solvent system H1 (EtOAc:acetone:ethanol:water 4:1:1:1) in ethyl acetate in the form of white amorphous solid.
~ 1:1 mixture of diastereoisomers

^{1}H NMR (500.0 MHz, CD_3OD): 0.90 (m, 6H, $C\mathbf{H_3}(CH_2)_{14}CH_2O$); 1.24-1.45 (m, 52H, $CH_3(C\mathbf{H_2})_{13}CH_2CH_2O$); 1.66-1.72 (m, 4H, $CH_3(CH_2)_{13}C\mathbf{H_2}CH_2O$); 2.10-2.20 (m, 4H, $PC\mathbf{H_2}CH_2N$); 2.62-2.66 (m, 6H, HC≡C, H-2b,5b-pyrrolidine); 2.82 – 2.92 (m, 4H, $NC\mathbf{H_2}CH_2P$); 3.04-3.10 (m, 4H, H-2a,5a-pyrrolidine); 4.03 – 4.20 (m, 18H, H-2′,3′,4′, H-3,4-pyrrolidine, $CH_3(CH_2)_{14}C\mathbf{H_2}O$, CH_2NH); 4.25 – 4.44 (m, 4H, H-5′); 5.82, 5.83 (2 × d, 2 × 1H, *J* = 7.0, H-1′); 5.91, 5.92 (2 × d, 2 × 1H, *J* = 7.5, H-5); 7.72, 7.75 (2 × d, 2 × 1H, *J* = 7.5, H-6).

^{13}C NMR (125.7 MHz, CD_3OD): 14.46 (**C**$H_3(CH_2)_{14}CH_2O$); 23.75 (CH_3(**C**$H_2)_{13}CH_2CH_2O$); 24.97, 25.03 (d, $J_{C,P}$ = 139.8, P**C**H_2CH_2N); 26.65, 30.28, 30.49, 30.68, 30.72, 30.74, 30.78, 30.81 (CH_3(**C**$H_2)_{13}CH_2CH_2O$, CH_2N); 31.55, 31.56 (d, $J_{C,P}$ = 5.9, $CH_3(CH_2)_{13}$**C**H_2CH_2O); 33.09 (CH_3(**C**$H_2)_{13}CH_2CH_2O$); 50.74, 50.76 (N**C**H_2CH_2P); 60.99, 61.00 (CH_2-2,5-pyrrolidine); 66.32, 66.43(d, $J_{C,P}$ = 6.1, CH_2-5′); 67.61, 67.68 (d, $J_{C,P}$ = 7.1, $CH_3(CH_2)_{13}CH_2$**C**H_2O); 70.55; 70.57 (CH-3′); 72.72 (H**C**≡C-); 75.57; 75.60 (CH-2′); 78.34; 78.40 (CH-3,4-pyrrolidine); 80.31 (-**C**≡CH); 83.13, 83.18 (d, $J_{C,P}$ = 6.2, CH-4′); 93.22, 93.24 (CH-1′); 96.82 (CH-5); 141.80; 141.87 (CH-6); 158.47, 158.48 (C-2); 165.04 (C-4).
$^{31}P\{^1H\}$ NMR (202.3 MHz, CD_3OD): 30.76, 31.04.
HRMS (ESI+) for $C_{34}H_{59}N_4O_9PNa$ $(M+Na)^+$: calcd 721.39119, found 721.39108

Synthesis biotinylated LPPO DR5690

Azide-PEG3-Biotin Conjugate was purchased from Jena Bioscience (JB) and used according to JB recommendation for the reaction with **DR5557**. The final **DR5690** was purified using preparative HPLC on reversed phase and its structure was proven by LC-MS.

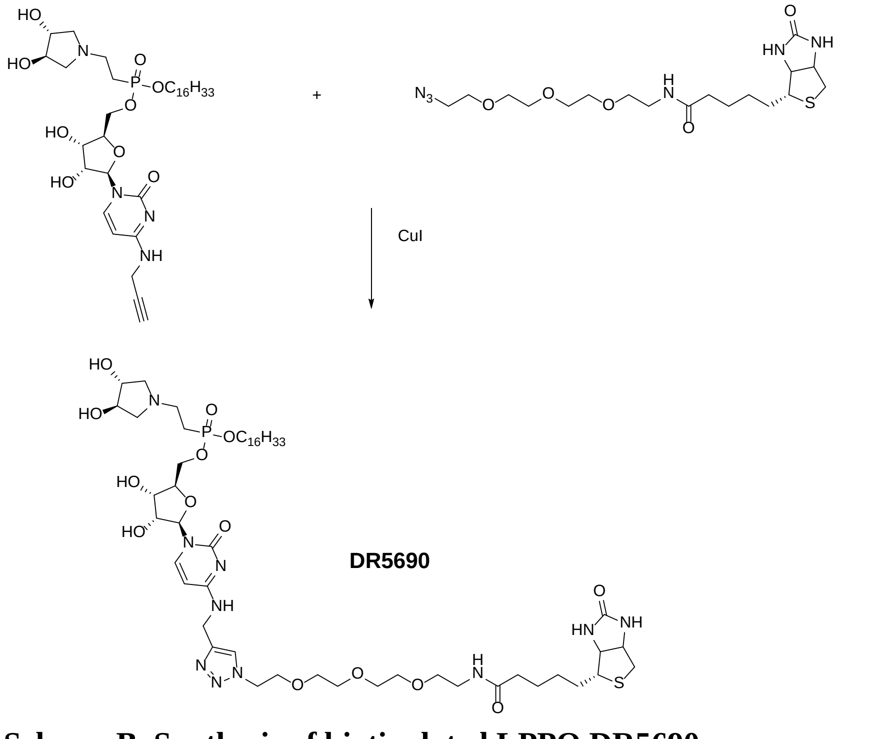

Scheme B. Synthesis of biotinylated LPPO DR5690.

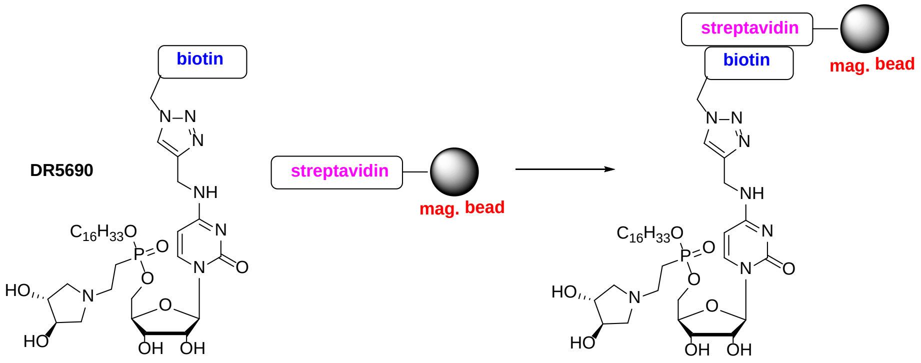

Scheme C. Preparation of magnetic beads with attached LPPO.

Synthesis of fluorescently labeled LPPO DR5823

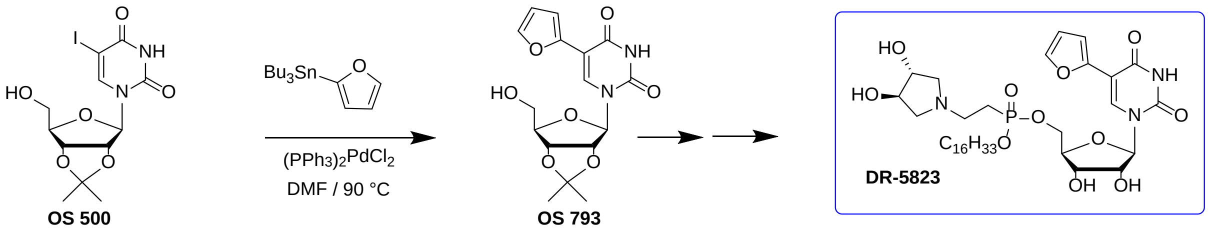

Scheme D. Synthesis of fluorescent LPPO DR5823.

The title compound was prepared according to described general procedure for LPPO synthesis [1] (same as for the compound **DR-5557** *vide infra*) in 45% yield from compound **OS793**.
DR-5823

~ 1:1 mixture of distrereoisomers

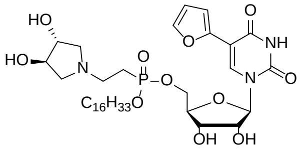

^{1}H NMR (500.0 MHz, DMSO-d_6): 0.90 (m, 6H, C**H$_3$**(CH$_2$)$_{14}$CH$_2$O); 1.18 – 1.38 (m, 52H, CH$_3$(C**H$_2$**)$_{13}$CH$_2$CH$_2$O); 1.61 - 1.68 (m, 4H, CH$_3$(CH$_2$)$_{13}$C**H$_2$**CH$_2$O); 2.21 - 2.36 (m, 4H, PC**H$_2$**CH$_2$N); 2.87 – 2.93 (m, 4H, H-2b,5b-pyrrolidine); 3.08 – 3.19 (m, 4H, PCH$_2$C**H$_2$**N); 3.22 – 3.29 (m, 4H, H-2b,5b-pyrrolidine); 4.05 – 4.15 (m, 5H, H-3,4-pyrrolidine, CH$_3$(CH$_2$)$_{13}$CH$_2$C**H$_2$**O); 4.17 – 4.21 (m, 2H, H-4′); 4.22, 4.23 (2 × t, 2 × 1H, $J_{3',2'} = J_{3',4'} = 5.5$, H-3′); 4.28, 4.30 (2 × dd, 2 × 1H, $J_{2',3'} = 5.3$, $J_{2',1'} = 4.2$, H-2′); 4.34 (ddd, 2H, $J_{gem} = 11.5$, $J_{H,P} = 6.7$, $J_{5'b,4'} = 3.9$, H-5′b); 4.38

(dd, 4H, $J_{H,P}$ = 6.2, $J_{5',4'}$ = 3.3, H-5′); 4.42 (ddd, 2H, J_{gem} = 11.5, $J_{H,P}$ = 5.5, $J_{5'a,4'}$ = 2.5, H-5′a); 5.92, 5.93 (2 × d, 2 × 1H, $J_{1',2'}$ = 4.2, H-1′); 6.50, 6.51 (2 × dd, 2 × 1H, $J_{4,3}$ = 3.3, $J_{4,5}$ = 1.8, H-4-furyl); 6.97 – 6.98 (m, 2H, H-3-furyl); 7.54 (dd, 2H, $J_{5,4}$ = 1.8, $J_{5,3}$ = 0.9, H-5-furyl); 8.03, 8.04 (2 × s, 2 × 1H, H-6).
^{13}C NMR (125.7 MHz, DMSO-d_6): 14.46 (**C**$H_3(CH_2)_{14}CH_2O$); 24.26, 24.29 (2 × d, $J_{C,P}$ = 140.2, P**C**H_2CH_2N); 23.74, 26.55, 26.58, 30.23, 30.24, 30.48, 30.62, 30.63, 30.67, 30.69, 30.76, 30.77, 30.79, 30.80 (CH_3(**C**$H_2)_{13}CH_2CH_2O$); 31.50 (d, $J_{C,P}$ = 6.0, $CH_3(CH_2)_{13}$**C**H_2CH_2O); 33.07 (CH_3(**C**$H_2)_{13}CH_2CH_2O$); 51.40, 51.41 (PCH_2**C**H_2N); 60.86, 60.88 (CH_2-2,5-pyrrolidine); 66.48 (d, $J_{C,P}$ = 6.3, CH_2-5′); 66.59 (d, $J_{C,P}$ = 5.9, CH_2-5′); 67.91, 67.97 (2 × d, $J_{C,P}$ = 7.0, $CH_3(CH_2)_{14}$**C**H_2O); 70.72, 70.79 (CH-3′); 75.09, 75.12 (CH-2′); 77.27 (CH-3,4-pyrrolidine); 83.72, 83.78 (2 × d, $J_{C,P}$ = 5.4, CH-4′); 92.09, 92.24 (CH-1′); 108.14, 108.15 (C-5); 110.08, 110.10 (CH-3-furyl); 112.73, 112.77 (CH-4-furyl); 135.60, 135.77 (CH-6); 142.67, 142.68 (CH-5-furyl); 147.49, 147.50 (C-2-furyl); 151.30, 151.31 (C-2); 162.11, 162.13 (C-4).
^{31}P{^{1}H} NMR (202.3 MHz, DMSO-d_6): 30.54, 30.62.
IR ν_{max}($CHCl_3$) 3352 (m, vbr), 2957 (m), 2926 (vs), 2854 (m), 1696 (vs), 1570 (w), 1500 (w), 1467 (m), 1380 (m), 1284 (m), 1268 (m), 1236 (m), 1153 (w), 1101 (m), 1077 (m, sh), 1051 (m), 1031 (m), 1000 (m), 885 (w), 820 (w), 661 (vw).
HRMS (ESI+) for $C_{35}H_{59}N_3O_{11}P$ $(M+H)^+$: calcd 728.38817, found 728.38724.

5-(furan-2-yl)-2´,3´-O-isopropyliden-uridine OS793 [2]
Bis(triphenylphosphine)palladium(II) dichloride (60 mg; 85 μmol; 5% mol) was added to the solution of 5-iodo-2´,3´-*O*-isopropylidene-uridine (0.7 g; 1.7 mmol) and 2-(tributylstannyl)furane (1.075 mL; 3.4 mmol) in DMF (10 mL) and reaction mixture was heated to 90 °C for 3 h. The reaction was then evaporated, dissolved in toluene and passed through Celite. Final **OS793** was obtained by the chromatography on silica gel using linear gradient of ethyl acetate in toluene in the form of white amorphous solid (380 mg; 64 %).

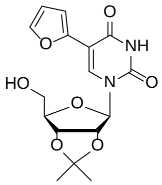

OS964

^{1}H NMR (600.1 MHz, DMSO-d_6): 1.30, 1.50 (2 × s, 2 × 3H, CH_3); 3.60 (ddd, 1H, J_{gem} = 11.7, $J_{5'b,OH}$ = 5.1, $J_{5'b,4'}$ = 4.5, H-5′b); 3.64 (ddd, 1H, J_{gem} = 11.7, $J_{5'a,OH}$ = 5.1, $J_{5'a,4'}$ = 4.1, H-5′a); 4.15 (ddd, 1H, $J_{4',5'}$ = 4.5, 4.1, $J_{4',3'}$ = 3.4, H-4′); 4.79 (dd, 1H, $J_{3',2'}$ = 6.3, $J_{3',4'}$ = 3.4, H-3′); 4.97 (dd, 1H, $J_{2',3'}$ = 6.3, $J_{2',1'}$ = 2.6, H-2′); 5.15 (t, 1H, $J_{OH,5'}$ = 5.1, OH-5′); 5.95 (d, 1H, $J_{1',2'}$ = 2.6, H-1′); 6.52 (dd, 1H, $J_{4,3}$ = 3.3, $J_{4,5}$ = 1.8, H-4-furyl); 6.86 (dd, 1H, $J_{3,4}$ = 3.3, $J_{3,5}$ = 0.9, H-3-furyl); 7.63 (dd, 1H, $J_{5,4}$ = 1.8, $J_{5,3}$ = 0.9, H-5-furyl); 8.26 (s, 1H, H-6); 11.70 (s, 1H, NH).
^{13}C NMR (150.9 MHz, DMSO-d_6): 25.33, 27.19 (CH_3); 61.39 (CH_2-5′); 80.67 (CH-3′); 84.24 (CH-2′); 87.03 (CH-4′); 91.86 (CH-1′); 105.72 (C-5); 108.25 (CH-3-furyl); 111.74 (CH-4-furyl); 113.02 (**C**$(CH_3)_2$); 136.11 (CH-6); 141.80 (CH-5-furyl); 146.48 (C-2-furyl); 149.49 (C-2); 160.37 (C-4).

HRMS (ESI-) for $C_{16}H_{17}O_7N_2$ $(M-H)^-$: calcd 349.10412, found 349.10391
IR $\nu_{max}(CHCl_3)$ 3627, 3459, 3387, 3193, 3074, 2963, 2874, 1712, 1699, 1571, 1500, 1465, 1438, 1385, 1378, 1279, 1155, 1122, 1082, 1075, 886, 854, 512.
