# Supplementary material for: Insights into the Mechanism of Action of Bactericidal Lipophosphonoxins
Source: PLoS One. 2015 Dec 30;10(12):e0145918. doi: 10.1371/journal.pone.0145918 (PMC4696656; doi:10.1371/journal.pone.0145918)
Supplement: S2 Supporting Information — (PDF) [file pone.0145918.s006.pdf]

MD simulations: methodology

Initial 55-ns MD simulation used a bacterial cell membrane mimic with 1-palmitoyl-2-oleoyl-*sn*-glycero-3-phosphoethanolamine (POPE) and 1-palmitoyl-2-oleoyl-*sn*-glycero-3-phospho-rac-(1-glycerol) (POPG) phospholipids (128xPOPE+24xPOPG). Eight **DR5026** molecules were positioned symmetrically relative to the POPE:POPG phospholipidic bilayer (four **DR5026** molecules were situated above and four bellow the membrane). Further, a small pore was pre-formed in the POPG membrane consisting of 82 phospholipids. Four **DR5026** were placed on each side of the pore. Nucleoside modules of LPPOs were directed inside the pore. Subsequently, a total of ten 50-ns MD runs were produced. Initially, all model membranes were built and equilibrated using CHARMM-GUI [1, 2]. Further, water molecules and ions were stripped from the CHARMM-GUI membranes. After the addition of **DR5026,** the simulated systems were resolvated and ionized in VMD [3]. The TIP3P water model [4] was used with the CHARMM36 lipid force field [5]. The **DR5026** force constants were obtained using the ParamChem web server [6]. All MD simulations were produced by means of the NAMD software package [7]. Simulated systems were energy-minimized using the steepest descent method for 1000 iterations. MD runs were carried out in the NPT ensemble using cubic periodic boundary conditions. Electrostatic interactions were calculated using the particle mesh Ewald (PME) method [8]. The Langevin thermostat [9] (without coupling to hydrogens and with a damping coefficient of 5/ps) was used for temperature control set at physiological conditions (310 K). The Langevin barostat [10] with an oscillation period of 100 fs and a decay of 50 fs was used to maintain the pressure at 1 atm. The VMD software package [3] was used for rendering snapshots from MD trajectories.

MD simulations: Results

To gain additional insights into the interaction of LPPOs with the plasmatic membrane, we performed molecular dynamics (MD) simulations.

At the beginning, **DR5026** molecules were placed in water outside the mixed POPE:POPG (1-palmitoyl-2-oleoyl-*sn*-glycero-3-phosphoethanolamine (POPE) and 1-palmitoyl-2-oleoyl-*sn*-glycero-3-phospho-rac-(1-glycerol) (POPG)) bilayer mimicking a bacterial membrane (S5 Fig – time 0 ns). Already after approx. 25 ns, all LPPOs found their way into the interior of the phospholipid bilayer (S5 Fig – time 25 ns). The high affinity of **DR5026** for the model bacterial membrane seemed to arise from electrostatic interactions between the cationic iminosugar modules of LPPOs and the head groups of anionic POPG lipids. Moreover, the hydrophobicity of lipophilic alkyl chains of LPPOs as well as the hydrophobicity of parts of uracil bases from nucleoside modules of LPPOs drove **DR5026** (with the exception of their iminosugar modules) deeper into the lipid tail region of the POPE:POPG membrane. It resulted in an amphiphilic positioning of **DR5026** with the cationic iminosugar modules located at the lipid-water interface where they were anchored towards the POPE:POPG anionic head groups (S5 Fig – time 55 ns).

As simulations at the time scale performed by the available technology (tens of nano seconds) did not allow to capture pore formation because this likely takes hundreds of nanoseconds to microseconds, we pre-formed a small pore in the model POPG membrane filled with water

molecules. Based on our experimental data, we placed four LPPOs with nucleoside modules directed inside the pore. We performed a total of ten MD simulations. About half of the pores survived 50 ns MD runs. The experimental data above (i.e. Hill coefficients indicating that at least four molecules are involved in forming the pore in vesicles) best corresponded with the pore formed in the MD run no. 1 (S6 Fig). The POPG membrane was remarkably curved in the vicinity of the pore (it is apparent from positions of phosphorus atoms of POPG head groups). Several POPG molecules even penetrated into the pore. Uracil bases of two **DR5026** molecules (anchored toward the opposite leaflets of the POPG membrane) directly interacted one with another. Two or three other **DR5026** molecules supported the pore stability. Water molecules formed a continuous chain passing from one side of membrane to the other. It is conceivable that such pores may adversely affect the integrity of the membrane.

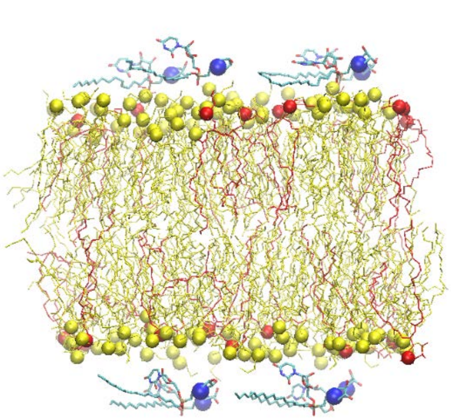

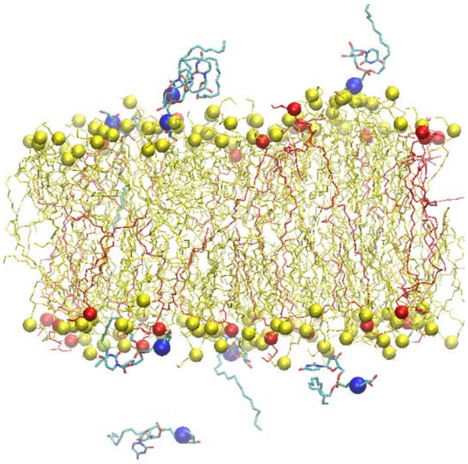

t = 0 ns **t = 5 ns**

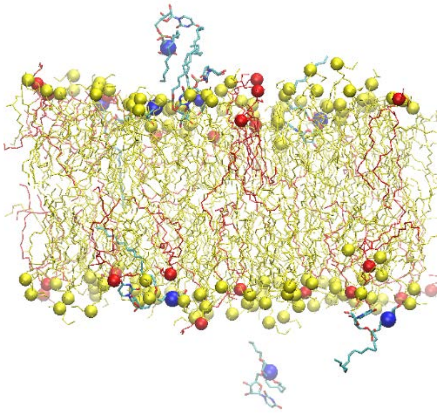

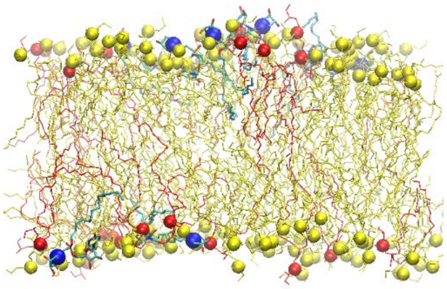

t = 15 ns **t = 25 ns**

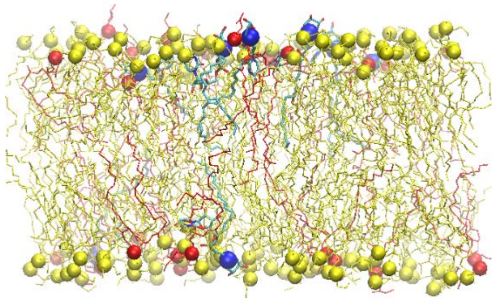

t = 55 ns

Figure A. Timeline of penetration of DR-5026 into the model POPE:POPG membrane. At the beginning, all **DR-5026** molecules were placed in water outside the mixed POPE:POPG bilayer mimicking a bacterial membrane. Already after approx. 25 from 55 ns of MD simulation, all **DR-5026** found their way into the interior of the phospholipid bilayer. The nitrogen atoms from the

iminosugar modules of **DR-5026** are highlighted as blue spheres. Phosphorus atoms of POPEs/POPGs are depicted as yellow/red spheres.

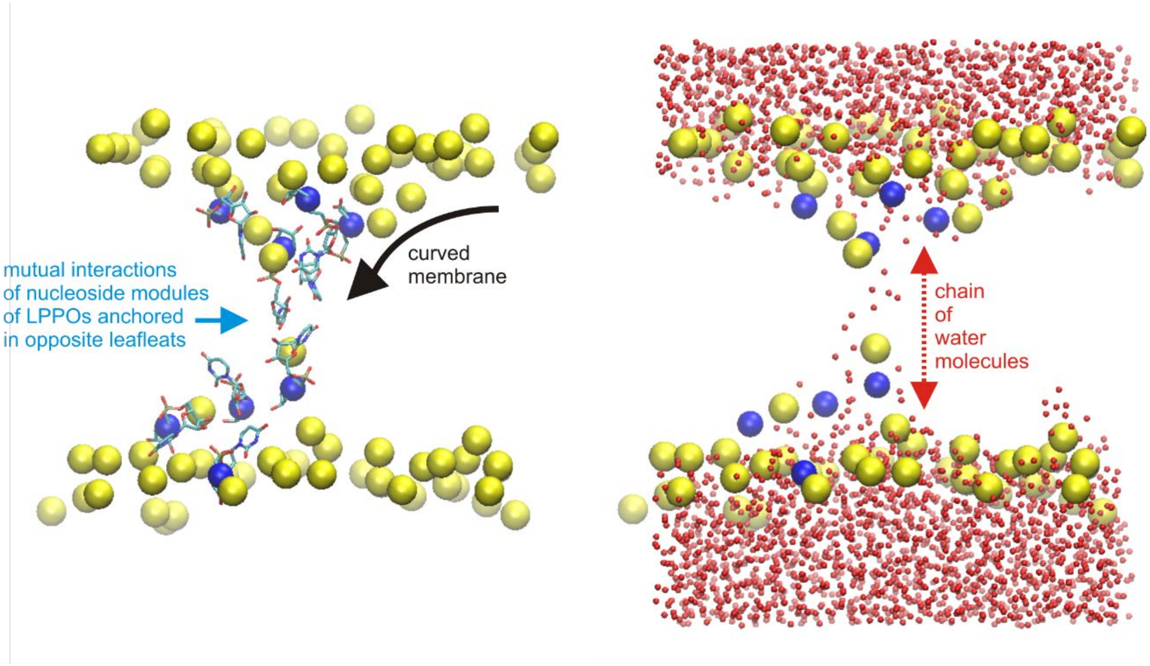

Figure B. A small pore was pre-formed and filled with water molecules in the model POPG membrane. Four **DR5026** with nucleoside modules directed inside the pore were placed on each side of the pore. The final snapshot of the 50 ns MD run no. 1, which corresponds best with the experimental data, is shown here. The nucleoside, iminosugar and phosphate modules of **DR5026** are depicted using sticks. The nitrogen atoms from the iminosugar modules of **DR5026** are highlighted as blue spheres. The phosphorus atoms of POPGs are depicted as large yellow spheres. Solvent oxygen atoms are shown as small red spheres. The hydrophobic modules of LPPOs and almost all atoms of POPGs are hidden for clarity.
