# Supplementary material for: Insights into the Mechanism of Action of Bactericidal Lipophosphonoxins
Source: PLoS One. 2015 Dec 30;10(12):e0145918. doi: 10.1371/journal.pone.0145918 (PMC4696656; doi:10.1371/journal.pone.0145918)
Supplement: S3 Supporting Information — (PDF) [file pone.0145918.s007.pdf]

S3 Supporting information. MTD test in mouse model – a detailed description

The MTD (maximum tolerated dose) in mouse model was carried out by MediTox s.r.o. on the commercial base.

Regulatory Guidelines

The study was carried out according to:

ICH M3(R2) – The International Conference on Harmonization of Technical Requirements for Registration of Pharmaceuticals for Human Use. Relevant SOPs (Standard operating procedures) of MediTox s.r.o.

Facility Management and Animal Husbandry

Animal care was in compliance with the SOPs of MediTox s.r.o., the European convention for the protection of vertebrate animals used for experimental and other scientific purposes (ETS 123), the Czech Collection of laws No. 246/1992, inclusive of the amendments, on the Protection of animals against cruelty, and Public Notice of the Ministry of Agriculture of the Czech Republic, Collection of laws No. 419/2012 as amended, on keeping and exploitation of experimental animals. MediTox s.r.o. is a holder of the accreditation Certificate for user's issued by Central Committee for Animal Protection of the Czech Republic.

Animal Welfare Act Compliance

The study was prepared for this type of experiment and approved by the Institutional Animal Care and Use Committee (IACUC) and the Committee for Animal Protection of the Ministry of Health (PP 09/2013) of the Czech Republic. Procedures used in this report were designed to conform to accepted practices and to minimize or avoid causing pain, distress, or discomfort to the animals. The number of animals selected for use in this study was considered to be the minimum number necessary to meet scientific and regulatory guidelines for this type of study.

Preparation of the Test item application formulations

For the administration the appropriate amount of the Test item (TI) delivered to MediTox s.r.o. was dissolved in aqua for injection and orally (in the volume of 0.5 mL/25g of body weight) and intraperitoneally (in the volume of 1.0 mL/25g of body weight) administered to mice.

Test System

Table 1 Test system

Species & Strain:	Mice ICR
Supplier:	VELAZ – Charles River
Quality at delivery:	SPF
Age on delivery:	6 weeks
Age at treatment start:	More than 6 weeks
Body weight at delivery:	Males: 30-38 g<br>Females: 25-32 g
Number of animals per group:	6 (3 females + 3 males)
Number of groups:	10 (5 per tested way of administration)
Number of animals:	60 (30 females + 30 males)

Allocation and Dosing

Table 2 Allocation and dosing

Way of the administration	Group	Dose	No. of animals	
			Males	Females
Orally	D1-PO	5 mg/kg	M1, M2, M3	F4, F5, F6
	D2-PO	500 mg/kg	M7, M8, M9	F10, F11, F12
	D3-PO	1000 mg/kg	M13, M14, M15	F16, F17, F18
	D4-PO	1500 mg/kg	M19, M20, M21	F22, F23, F24
	D5-PO	2000 mg/kg	M25, M26, M27	F28, F29, F30
Intraperitoneally	D1-IP	5 mg/kg	M31, M32, M33	F34, F35, F36
	D2-IP	500 mg/kg	M37, M38, M39	F40, F41, F42
	D3-IP	250 mg/kg	M43, M44, M45	F46, F47, F48
	D4-IP	80 mg/kg	M49, M50, M51	F52, F53, F54
	D5-IP	50 mg/kg	M55, M56, M57	F58, F59, F60

Animal Selection, Randomization and Group Assignment

Animals were sorted according to body weight and allocated to the dose groups (SOP SN-TOX-00).

Justification of Dose Level and Administration Route

The first dose and route of administration to be used in this study was determined on the basis of the preliminary experiments.

Justification for the Test System

The mice are recognized by international guidelines as a recommended test system.

Housing

The study animals were housed in Macrolon Tecniplast cages (3 females/cage, males in individual cages) in monitored laboratory conditions at building No.2, part 2, room No. 5. Room temperature was 19-25 °C and the relative humidity was between 30-70%. Fluorescent lighting provided illumination 12 hours per day. Feed and water containers were changed and sanitized at least once weekly. Tier Wohl Super (JRS Germany) was used as bedding.

Diet

The animals were fed with standard pellet diet for rodents of monitored quality (analyzed 2 times per year for possible toxic or microbiological contamination - the certificates are available in MediTox s.r.o.archive) during the acclimation and study periods. Feed "Myši chov" (Sehnoutek a synové v.o.s., Czech Republic) was provided ad libitum. The diet did not contain any contaminants at levels that could reasonably be expected to affect the purpose or integrity of the study.

Water

Water of monitored quality (analyzed minimally 2 times per year for possible toxic or microbiological contamination, the certificates are available in MediTox s.r.o. archive) was supplied ad libitum during the acclimation and study periods. The water did not contain any contaminants at levels that could reasonably be expected to affect the purpose or integrity of the study.

Acclimation

Mice were acclimated for at least 7 days. No prophylactic or therapeutic treatment was administered during the acclimation period. Only animals in good health conditions were used for the study.

Animal Identification

The cages were marked with information about the Study No., dose group, animals No., and their branding. The animals were branded with tail tattoo according to SOP SN-TOX-00.

Dose Procedure

Each animal from the dose groups was administered orally (in the volume of 0.5 mL/25g of body weight) or intraperitoneally (in the volume of 1.0 mL/25g of body weight) by the determined dose of the Test item.

Six mice (3 females and 3 males) per group were dosed in stepwise procedure using fixed doses. The initial level was selected on the decision of Sponsor on the basis of previously available information (5 mg/kg of body weight).

Further groups of animals were dosed with higher or lower fixed doses, depending on the presence or absence of signs of toxicity or mortality during 48 hours after the administration. This procedure continued until the dose causing evident toxicity or no more than one death was identified. The time interval between dosing at each level was determined by the onset, duration, and severity of toxic signs. Treatment of animals at the next dose group was delayed until one was confident of survival of the previously dosed animals – after 48 hours.

The animals were observed after single administration for 7 days.

DAILY Clinical Observations

All animals from the study were observed daily for clinical signs, morbidity or mortality during acclimation, 0.5, 4, 6, 24 and 48 hours after administration. Onset, duration and severity of any signs were recorded, and used as criteria for early euthanasia decisions. The clinical observations included:

Changes in skin, fur, eyes, occurrence of secretion and excretion

Autonomic activity (e.g. lacrimation, piloerection)

Changes in gait and posture

Changes in unprovoked behavior, presence of stereotypes or bizarre behavior

Response to handling

Body Weight

All the mice were individually weighed during Week –1, before the administration and then before the necropsy.

Terminal observations

All the animals that died during study or survived to their scheduled euthanasia (cervical dislocation under ether anesthesia) 8th day after the administration received a complete post-mortem examination and microscopic examination of organs showing evidence of gross pathology.

Necropsy

All the animals were weighed and examined externally. The cranial, thoracic and visceral cavities were opened and examined macroscopically. Abnormalities in visceral cavities were recorded with details of location, color, shape and size. Livers were collected and preserved in 4 % neutral buffered formaldehyde.

Table Set II.

Pathology

LIST OF ABBREVIATIONS AND SYMBOLS

**D1-PO**	=	DR-5026 in dose of 5 mg/kg p.o.
**D2-PO**	=	DR-5026 in dose of 500 mg/kg p.o.
**D3-PO**	=	DR-5026 in dose of 1000 mg/kg p.o.
**D4-PO**	=	DR-5026 in dose of 1500 mg/kg p.o.
**D5-PO**	=	DR-5026 in dose of 2000 mg/kg p.o.
**D1-IP**	=	DR-5026 in dose of 5 mg/kg i. p.
**D2-IP**	=	DR-5026 in dose of 500 mg/kg i. p.
**D3-IP**	=	DR-5026 in dose of 250 mg/kg i. p.
**D4-IP**	=	DR-5026 in dose of 80 mg/kg i. p.
**D5-IP**	=	DR-5026 in dose of 50 mg/kg i. p.

Codes and symbols used at animal level

**M**	=	MALE
**F**	=	FEMALE

Codes and symbols used at finding level

**GRADE 1**	=	MINIMAL/VERY FEW/VERY SMALL
**GRADE 2**	=	SLIGHT/FEW/SMALL
**GRADE 3**	=	MODERATE/MODERATE NUMBER/MODERATE SIZE
**GRADE 4**	=	MARKED/MANY/LARGE
)	=	FINDING UNILATERAL IN PAIRED ORGANS
**P**	=	FINDING PRESENT, SEVERITY NOT SCORED
**NA**	=	NOT APPLICABLE

Table 3: Severity grade of gross pathology findings in male and female mice of the D1-PO group – individual animal data

**Dose group:**	**D1-PO**					
**Sex:**	**M**			**F**		
**Animal number:**	**M1**	**M2**	**M3**	**F4**	**F5**	**F6**
NO FINDINGS	-	-	-	-	-	-

Table 4: Severity grade of gross pathology findings in male and female mice of the D2-PO group – individual animal data

**Dose group:**	**D2-PO**					
**Sex:**	**M**			**F**		
**Animal number:**	**M7**	**M8**	**M9**	**F10**	**F11**	**F12**
NO FINDINGS	-	-	-	-	-	-

Table 5: Severity grade of gross pathology findings in male and female mice of the D3-PO group – individual animal data

**Dose group:**	**D3-PO**					
**Sex:**	**M**			**F**		
**Animal number:**	**M13**	**M14**	**M15**	**F16**	**F17**	**F18**
NO FINDINGS	-	-	-	-	-	-

Table 6: Severity grade of gross pathology findings in male and female mice of the D4-PO group – individual animal data

**Dose group:**	**D4-PO**					
**Sex:**	**M**			**F**		
**Animal number:**	**M19**	**M20**	**M21**	**F22**	**F23**	**F24**
NO FINDINGS	-	-	-	-	-	-

Table 7: Severity grade of gross pathology findings in male and female mice of the D5-PO group – individual animal data

**Dose group:**	**D5-PO**					
**Sex:**	**M**			**F**		
**Animal number:**	**M25**	**M26**	**M27**	**F28**	**F29**	**F30**
NO FINDINGS	-	-	-	-	-	-

Table 8: Severity grade of gross pathology findings in male and female mice of the D1-IPgroup – individual animal data

**Dose group:**	**D1-IP**					
**Sex:**	**M**			**F**		
**Animal number:**	**M31**	**M32**	**M33**	**F34**	**F35**	**F36**
NO FINDINGS	-	-	-	-	-	-

Table 9: Severity grade of gross pathology findings in male and female mice of the D2-IP group – individual animal data

**Dose group:**	**D2-IP**					
**Sex:**	**M**			**F**		
**Animal number:**	**M37***	**M38***	**M39**	**F40**	**F41***	**F42***
**Abdominal cavity**						
- red liquid	1	1	-	-	2	1
**Kidneys**						
- venostasis	2	2	-	-	3	2
**Liver**						
- venostasis	2	2	-	-	3	2
**Lungs**						
- focal hemorrhage	-	-	--	-	-	2

* deceased animal

Table 10: Severity grade of gross pathology findings in male and female mice of the D3-IP group – individual animal data

**Dose group:**	**D3-IP**					
**Sex:**	**M**			**F**		
**Animal number:**	**M43***	**M44+**	**M45+**	**F46***	**F47+**	**F48***
**Abdominal cavity**						
- red liquid	1	1	1	1	1	1
**Kidneys**						
- venostasis	2	2	2	3	2	2
**Liver**						
- venostasis	2	2	2	3	2	2
**Lungs**						
- venostasis	3	-	-	-	-	-
**Thoracic cavity**						
- red liquid	1	-	-	1	-	-

+ killed in a moribund state

* deceased animal

Table 11: Severity grade of gross pathology findings in male and female mice of the D4-IP group – individual animal data

**Dose group:**	**D4-IP**					
**Sex:**	**M**			**F**		
**Animal number:**	**M49**	**M50**	**M51+**	**F52**	**F53**	**F54**
**Abdominal cavity**						
- red liquid	-	-	1	-	-	-
**Kidneys**						
- venostasis	-	-	2	-	-	-
**Liver**						
- enlarged	1	1	-	1	1	1
- rounded edges	3	3	-	3	3	3
- venostasis	-	-	2	-	-	-

+ killed in a moribund state

Table 12: Severity grade of gross pathology findings in male and female mice of the D5-IP group – individual animal data

**Dose group:**	**D5-IP**					
**Sex:**	**M**			**F**		
**Animal number:**	**M55**	**M56**	**M57**	**F58**	**F59**	**F60**
**Liver**						
- peritoneal adhesions	-	3	3	-	-	-
- enlarged	1	1	2	1	1	1
- rounded edges	3	3	3	3	3	3
**Spleen**						
- peritoneal adhesions	-	3	3	-	3	-

Table 13: Gross pathology findings – Summary animal data

Dose group:	D1-		D2-		D3-		D4-		D5-		D1-		D2-		D3-		D4-		D5-	
**Sex :**	**M**	**F**	**M**	**F**	**M**	**F**	**M**	**F**	**M**	**F**	**M**	**F**	**M**	**F**	**M**	**F**	**M**	**F**	**M**	**F**
**Number of animals:**	**3**	**3**	**3**	**3**	**3**	**3**	**3**	**3**	**3**	**3**	**3**	**3**	**3**	**3**	**3**	**3**	**3**	**3**	**3**	**3**
**Organ findings:**																				
**Abdominal cavity**																				
- red liquid	-	-	-	-	-	-	-	-	-	-	-	-	2	2	3	3	1	-	-	-
**Kidneys**																				
- venostasis	-	-	-	-	-	-	-	-	-	-	-	-	2	2	3	3	1	-	-	-
**Liver**																				
- peritoneal adhesions	-	-	-	-	-	-	-	-	-	-	-	-	-	-	-	-	-	-	2	-
- enlarged	-	-	-	-	-	-	-	-	-	-	-	-	-	-	-	-	2	3	3	3
- rounded edges	-	-	-	-	-	-	-	-	-	-	-	-	-	-	-	-	2	3	3	3
- venostasis	-	-	-	-	-	-	-	-	-	-	-	-	2	2	3	3	1	-	-	-
**Spleen**																				
- peritoneal adhesions	-	-	-	-	-	-	-	-	-	-	-	-	-	-	-	-	-	-	2	1
**Lungs**																				
- focal hemorrhage	-	-	-	-	-	-	-	-	-	-	-	-	-	1	-	-	-	-	-	-
- venostasis	-	-	-	-	-	-	-	-	-	-	-	-	-	-	1	-	-	-	-	-
**Thoracic cavity**																				
- red liquid	-	-	-	-	-	-	-	-	-	-	-	-	-	-	1	1	-	-	-	-
