# Supplementary material for: Insights into the Mechanism of Action of Bactericidal Lipophosphonoxins
Source: PLoS One. 2015 Dec 30;10(12):e0145918. doi: 10.1371/journal.pone.0145918 (PMC4696656; doi:10.1371/journal.pone.0145918)

S4 Supporting information. LogD calculation.

Software name and version: ACD/Percepta 14.0.0 (Build 2726)

Compound name: DR5026

LogD at:

pH = 1,7 (Stomach): 0,61
pH = 4,6 (Duodenum): 0,91
pH = 6,5 (Jejunum and Ileum): 2,48
pH = 7,4 (Blood): 3,22
pH = 8,0 (Colon): 3,51

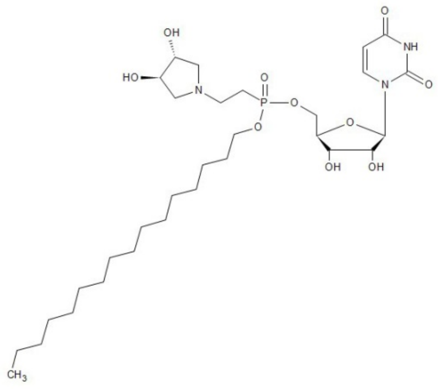

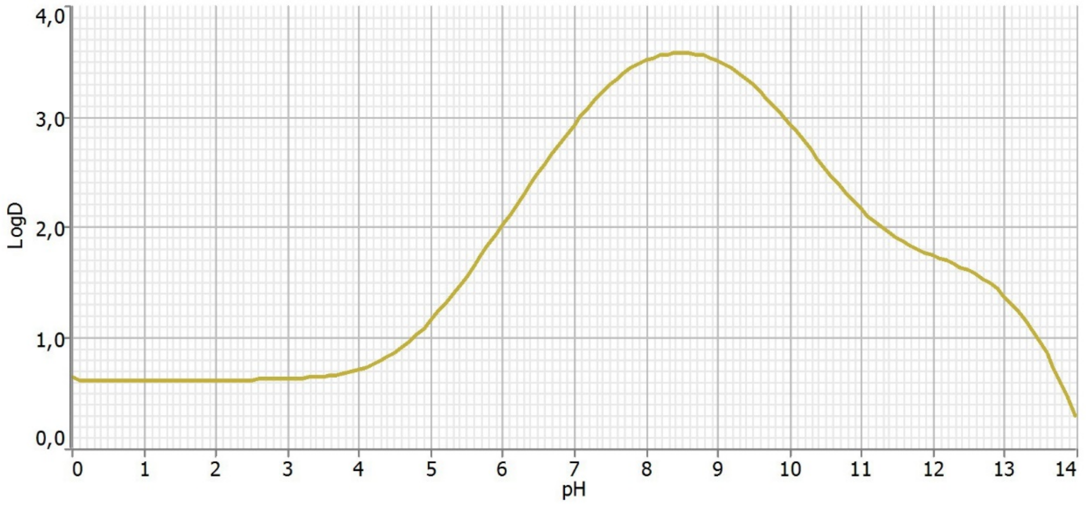

Compound name: DR5047

LogD at:

pH = 1,7 (Stomach): 0,73
pH = 4,6 (Duodenum): 1,03
pH = 6,5 (Jejunum and Ileum): 2,62
pH = 7,4 (Blood): 3,35
pH = 8,0 (Colon): 3,63

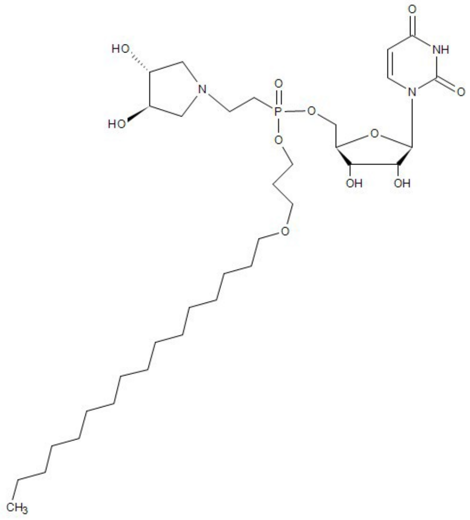

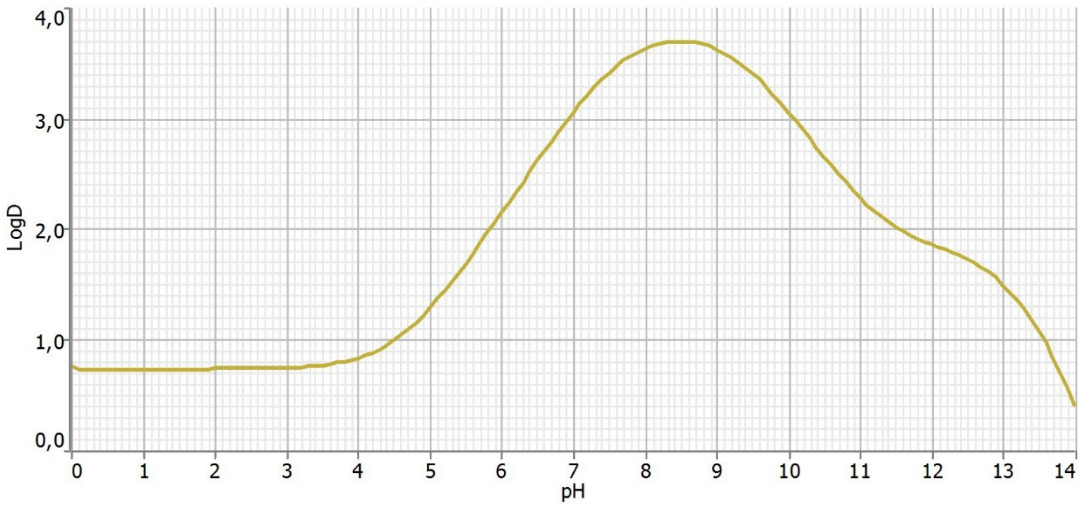

Supplement: S4 Supporting Information — (PDF) [file pone.0145918.s008.pdf]
