# Supplementary material for: Insights into the Mechanism of Action of Bactericidal Lipophosphonoxins
Source: PLoS One. 2015 Dec 30;10(12):e0145918. doi: 10.1371/journal.pone.0145918 (PMC4696656; doi:10.1371/journal.pone.0145918)
Supplement: S1 Table — (PDF) [file pone.0145918.s009.pdf]

S1 Table Antibacterial activity of LPPOs DR5557, DR5690, and DR5823

	MIC µg/ml		
	**DR5557**	**DR5690**	**DR5823**
Enterococcus faecalis CCM 4224	6.25	>200	6.25
Staphylococcus aureus CCM 4223	3.125	>200	25
Bacillus subtilis	3.125	50	12.5
Streptococcus agalactiae	6.25	12.5	3.125
S. aureus MRSA 4591	6.25	>200	25
S. haemolyticus 16568	6.25	>200	>200
E. faecium VanA 419/ana	25-12.5	>200	6.25
S. epidermidis 8700/B	3.125	25	6.25
