# Supplementary material for: Insights into the Mechanism of Action of Bactericidal Lipophosphonoxins
Source: PLoS One. 2015 Dec 30;10(12):e0145918. doi: 10.1371/journal.pone.0145918 (PMC4696656; doi:10.1371/journal.pone.0145918)
Supplement: S2 Table — Minimum inhibitory concentrations of the tested LPPOs after each induction step. The induction of resistance was carried out by repeated passages of E. faecalis and S. agalactiae strains with subinhibitory concentrations of the relevant LPPOs. A total of 14 passages were performed. The results clearly showed that the MICs remained relatively unchanged. It meant that the resistance was not induced. (PDF) [file pone.0145918.s010.pdf]

S2 Table. The induction of resistance of *Enterococcus faecalis* and *Streptococcus agalactiae* to compounds DR5047 and DR5026. Minimum inhibitory concentrations of the tested LPPOs after each induction step. The induction of resistance was carried out by repeated passages of *E. faecalis* and *S. agalactiae* strains with subinhibitory concentrations of the relevant LPPOs. A total of 14 passages were performed. The results clearly showed that the MICs remained relatively unchanged. It meant thatthe resistance was not induced.

Step	MIC (mg/L)			
	Streptococcus agalactiae		*Enterococcus faecalis* CCM 4224	
	**DR5026**	**DR5047**	**DR5026**	**DR5047**
1	3.1	6.2	6.2	12.5
2	3.1	6.2	6.2	12.5
3	3.1	6.2	6.2	12.5
4	3.1	6.2	6.2	12.5
5	3.1	6.2	6.2	25
6	3.1	6.2	12.5	25
7	6.2	3.1	12.5	25
8	6.2	6.2	12.5	25
9	3.1	6.2	12.5	25
10	3.1	6.2	12.5	50
11	3.1	6.2	12.5	25
12	6.2	6.2	12.5	25
13	6.2	6.2	12.5	25
14	6.2	6.2	12.5	25
